# Role of polymeric nanocomposite for tissue engineering applications

**DOI:** 10.1039/d6ra01646d

**Published:** 2026-06-05

**Authors:** Shivakumar Naik, Uma Palanikumar, Amgoth Ramesh, Udayakumar D, B. C. Revanasiddappa, Harishkumar Madhyastha, Akhil Nair, Wilona Loren Lobo, Pavithra Pradeep Prabhu

**Affiliations:** a Department of Chemistry, National Institute of Technology Karnataka Surathkal Mangalore-575025 Karnataka India udayakumar@nitk.edu.in udayaravi80@gmail.com; b Vidyothini Edutech Pvt. Ltd Tumkur Karnataka 561202 India; c Department of Plant Biotechnology, Center for Plant Molecular Biology and Biotechnology, Agricultural University Tamil Nadu India; d Nitte (Deemed to Be University), NGSM Institute of Pharmaceutical Sciences (NGSMIPS Department of Pharmaceutical Chemistry Mangalore India revan@nitte.edu.in; e Department of Cardiovascular Physiology, University of Miyazaki Miyazaki Japan; f Nitte (Deemed to Be University), NGSM Institute of Pharmaceutical Sciences (NGSMIPS), Department of Pharmaceutics Mangalore India; g Nitte (Deemed to Be University), NGSM Institute of Pharmaceutical Sciences (NGSMIPS), Department of Pharmacology Mangalore India; h Department of Pharmacognosy, Manipal College of Pharmaceutical Sciences, Manipal Academy of Higher Education Manipal Karnataka 576104 India pavithra.prabhu@manipal.edu

## Abstract

Polymeric nanocomposites have become revolutionary biomaterials for tissue engineering due to their unparalleled tunability by combining the design flexibility of polymeric matrices and functional properties of nano-fillers. This review provides a critical discussion of the principles of designing, synthesizing, fabricating, characterizing, and applying polymeric nanocomposites towards the tissue engineering purpose. The latest achievements in polymeric nanocomposites such as artificial intelligence-based scaffold design, clinical implementation of collagen-based nerve conduit, and novel stimuli-responsive 4D nanocomposites are analyzed in detail based on their importance to the field. Instead of a mere listing of materials, the review offers a comprehensive comparison between different approaches towards fabricating polymeric nanocomposites depending on their application towards different types of tissues, characterization, and clinical applications. The regulatory aspects and current barriers towards translating these materials into clinics are discussed as well. Finally, the review offers specific predictions about the three most promising polymer-nano filler combinations and three major challenges to this field within the next five years.

## Abstract 2

Polymeric nanocomposites have become revolutionary biomaterials for tissue engineering due to their unparalleled tunability by combining the design flexibility of polymeric matrices and functional properties of nano-fillers.

## Graphical abstract

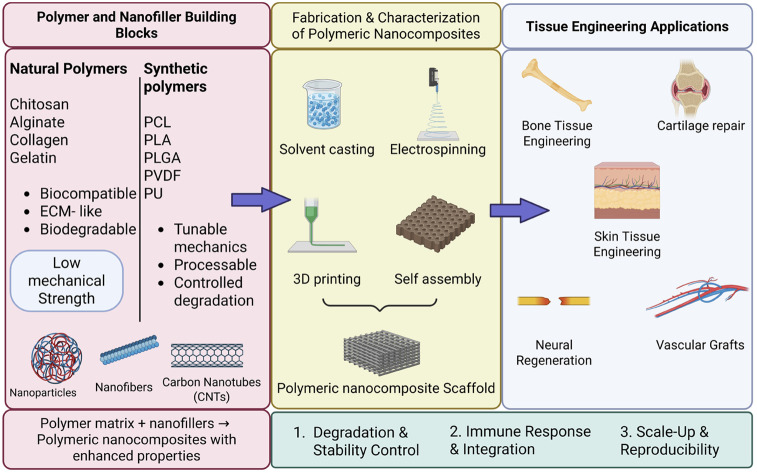

## 1. Introduction

One of the main problems associated with medicine is the problem of healing severely damaged tissues or organs due to injuries or degeneration. The conventional methods of grafting, like allografts, autografts, and biomedical implants, have many disadvantages like donor scarcity, risks of infections, poor biocompatibility with host tissues, and potential implant rejection. However, tissue engineering and regenerative medicine provide an alternative method for replacing the damaged tissues with those made in the laboratory using the principles of engineering and biology. These advanced strategies have shown promising results in helping the body repair and regenerate different types of tissues, such as bone, cartilage, blood vessels, and skin.^1^ In tissue engineering, the design of scaffolds should meet certain strict demands in regard to mechanical and biological characteristics. The three basic elements include (i) guiding scaffolds for tissue engineering; (ii) source of cells; and (iii) proper environment containing certain stimulating factors.^2^

**Fig. 1:**
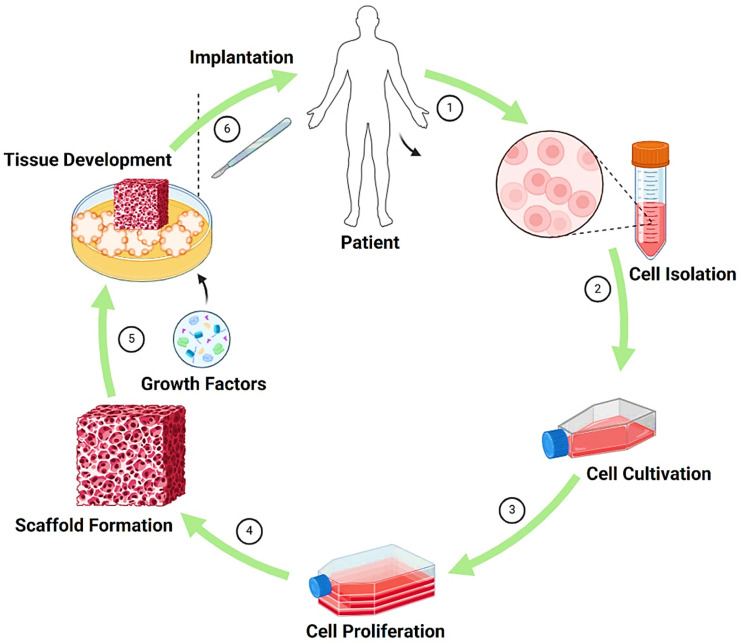
Key steps in tissue engineering: (1) implantation, (2) cell isolation, (3) cell cultivation, (4) cell proliferation, (5) scaffold formation, and (6) tissue development for regeneration. The images were created with Biorender.com.

As shown in Fig. 1 below, tissue engineering involves a sequence of steps whereby cells are extracted from the body of the recipient and developed into a viable tissue substitute that is fit for transplantation. In Step 1, cell isolation, cells are harvested from either the body of the recipient or the body of another individual who serves as a good donor through a minimal biopsy. These cells can be autologous, allogeneic, syngeneic, or xenogeneic in nature, and once isolated, they are then cultured or grown *in vitro*.^3^ During cell cultivation (Step 2), the isolated cells are kept in a nutrient-rich environment where temperature, oxygen level, and media composition are carefully maintained so the cells stay healthy and retain their original identity. As cells begin to cover the culture surface in cell proliferation (Step 3), the degree of surface coverage known as confluence becomes a critical factor, since as cells approach full confluence, their metabolism and proliferation behaviour begin to change.^4,5^ The proliferated cells are then placed onto a three-dimensional support structure in scaffold formation (Step 4); this scaffold acts as a host material for the seeded cells, guiding their attachment, growth, differentiation, proliferation, and migration, while the cells overall behaviour and fate remain closely tied to how and when the scaffold breaks down during tissue development.^6,7^ In step 5 during growth factors, signaling proteins like VEGF, FGF, and TGF-β are introduced into the cell-seeded scaffold; the proliferation and differentiation of progenitor cells and stem cells are triggered by growth factors, which are involved in wound healing, angiogenesis, and morphogenesis, requiring that their controlled release be a key component in directing cells to the desired tissue type. Free growth factors have poor stability within the body, where they quickly become inactive, so they are often entrapped within a scaffold structure to provide sustained release.^8^ Lastly, the developed construct is implanted into the damaged area using a process known as implantation (Step 6), and in this stage, the scaffold should not elicit any detrimental immune reactions, but rather, it should effectively interact with the biological surroundings in such a way that the cells can attach, proliferate, and differentiate, and in doing so, no cytotoxicity or inflammatory reactions are created as the scaffold slowly degrades.^9^ This combination of cells, scaffolds, and growth factors constitutes the backbone of tissue engineering, offering a solution to create or replace tissues and organs that can fulfill the increasing demand from a very small supply due to conventional donations.^10^

Conventional scaffold materials made from either a pure polymer or a single ceramic have a major limitation: they can rarely satisfy all the mechanical, biological, and degradation requirements of a target tissue at the same time. Polymer nanocomposites were developed as a direct answer to this problem. A polymer nanocomposite is a material in which tiny particles, fibres, or tubes measured at the nanoscale typically between 1 and 100 nanometres in at least one dimension are distributed throughout a polymer backbone to produce a new material whose combined properties are far superior to those of either component alone.^11^ Because nanoscale fillers have a much larger surface area than conventional micro-sized additives, they form a tighter and more intimate contact with the surrounding polymer chains, and this close interaction is what drives the substantial improvements seen in mechanical strength, electrical behaviour, and the ability of the surface to attract and retain proteins a prerequisite for cell adhesion, proliferation, and differentiation (https://www.intechopen.com/chapters/80617). Nanocomposites and nanomaterials are considered a strong platform for tissue engineering precisely because their structure can be made to resemble the natural extracellular matrix from the nanometre to the macroscopic scale, and this hierarchical resemblance is what ensures robust tissue formation by providing cells with the spatial and mechanical cues they expect to find in a living tissue.^12^ Adding nanomaterials such as carbon nanotubes, graphene oxide, titania nanotubes, or mesoporous silica into polymer matrices has also been shown to unlock entirely new functional properties including magnetic, electrical, and shape-memory behaviours that cannot be obtained from the polymer alone, opening the door to smart scaffolds that respond to external stimuli such as heat, light, or magnetic fields in the context of tissue repair.^13^ Taken together, these advantages explain why polymer nanocomposites have rapidly become the material of choice for scaffold fabrication across a wide range of tissue engineering applications, including bone, cartilage, skin, neural, and vascular tissue. The growing interest in polymer nanocomposites for these applications forms the central focus of the present review.

This review is motivated by a specific gap in the existing literature. While several reviews have addressed polymeric nanocomposites in tissue engineering from materials classification perspectives,^14–16^ none has simultaneously: (i) provided a critically comparative analysis across fabrication methods and tissue types; (ii) systematically addressed the clinical translation and regulatory landscape as of 2025–2026; and (iii) offered a rigorous analytical treatment of AI/ML integration in scaffold design. This review addresses this need. Additionally, the period 2025–2026 is a watershed one, as the US Food and Drug Administration has provided new guidelines for the use of nanotechnology in medical devices in 2023, the first AI-based scaffold has reached the first phase of clinical trials in 2024, and 4D bioprinting platforms are commercially available. This review presents these advances within a critical analysis framework aimed at bridging materials science and clinical translation from “what was created” to “what is possible.” The rest of this review paper will be structured as follows: The second section deals with the classification of polymers used in nanocomposites and the modes of reinforcement with critical comparisons. The third section discusses the four most common processes used for the fabrication of these materials. The fourth section highlights the importance of characterization methods. The fifth section covers the various tissue-specific applications of the polymeric nanocomposites along with critical limitations of each of them. The sixth section discusses issues and challenges ahead, which include responsive materials, integration of AI/ML, translational medicine aspects, and regulation issues. Section 7 gives detailed conclusions based on the findings.

## 2. Classification of polymeric nanocomposites

**Fig. 2:**
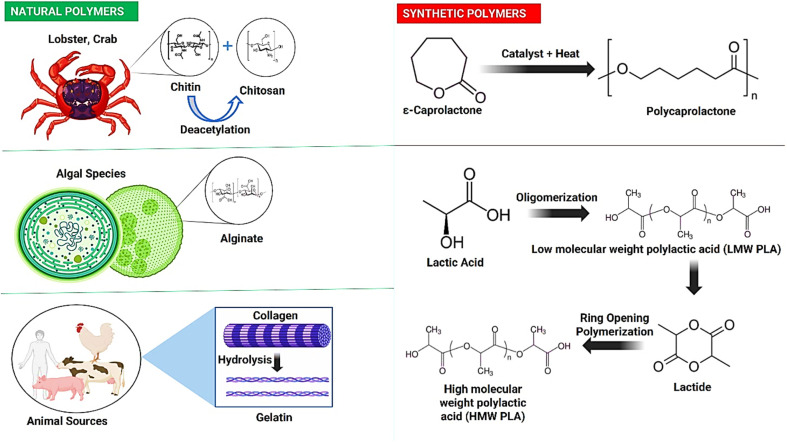
Classification of biomaterials: natural polymers (chitin, alginate, gelatin) *vs.* synthetic polymers (polycaprolactone, polylactic acid).

Choosing the right polymeric nanocomposite for a tissue engineering scaffold is not possible without first understanding how these materials are classified. At the broadest level, polymers are divided into natural and synthetic categories, each with distinct advantages and limitations for biological applications. Natural polymers can be further divided into polysaccharide-based and protein-based types, while synthetic polymers are grouped into biodegradable and non-biodegradable varieties.^16^ In addition to polymer type, nanocomposites are also classified by the kind of reinforcing nanomaterial added whether nanoparticles, nanofibers, or nanotubes because the geometry and chemistry of the filler directly determine the mechanical, electrical, and biological performance of the resulting scaffold.^17^Fig. 2 provides a visual overview of how biomaterials are classified in this context.

### 2.1 Based on polymer type

#### 2.1.1 Natural polymers

Natural polymers originate from biological systems such as plants, animals, and microorganisms and are synthesised through the same metabolic pathways that build the body's own structural and functional molecules. Their most important practical advantage is that their chemical structure closely resembles molecules already present in human tissues, which gives them inherent biocompatibility and reduces the risk of harmful immune responses.^18^ They are also degraded by enzymes that exist naturally in the body, meaning their breakdown can be managed through changes in structure and crosslinking density rather than synthetic chemistry. Nonetheless, natural polymers exhibit some common disadvantages that appear in literature independently of the tissue type. Firstly, these polymers possess poor mechanical strength. Secondly, these polymers show different properties depending on the batch and biological source. Thirdly, there is a chance that these polymers contain biological impurities such as endotoxins, proteins, and heavy metals that cause immune response when processed.^16^ All the above-mentioned shortcomings account for the absence of usage of natural polymers as a monomer material in tissue engineering. Instead, natural polymers are used in combination with other polymers or fillers. In accordance with their chemical structure and biological source, natural polymers in tissue engineering can be divided into two major categories: polysaccharides and proteins.^19^

##### 2.1.1.1 Polysaccharide-based polymers

Chitin is the second most abundant natural polymer found in nature after cellulose. Chitin comprises the hard structure of crustaceans, insects, and fungi cell walls, and it is produced from uridine diphosphate *N*-acetyl-d-glucosamine through chitin synthase enzymes.^20–22^ This biopolymer is structurally robust, partly degradable, and possesses weak immunological activity; thus, chitin can be used to manufacture porous sponges, hydrogels, and nanofibers for biomedical applications. The major disadvantage associated with chitin's direct application is its insolubility in water and most solvents.

Chitosan is obtained from chitin by de-acetylation, which leads to the formation of free amino (–NH_2_) groups within the polymer backbone.^21,22^ The presence of amino groups gives chitosan its unique characteristics, which include solubility in acidic aqueous media following protonation, and a positively charged surface due to electrostatic interactions with the negatively charged elements in the ECM, like glycosaminoglycans. What makes chitosan special compared to other biomaterials is its excellent biocompatibility, biodegradability, and adaptability in structure that allows it to form nanoparticles, scaffolds, hydrogels, membranes, and films. It also has broad-spectrum antimicrobial capabilities that are based on its ability to disorganize cell membranes, which is an essential feature in biomedical applications such as wound healing and bone tissue engineering in which controlling infections is of paramount importance.^23,24^ Chitosan scaffolds have been shown to exhibit osteoinductive and osteoconductive behaviour when applied in bone tissue engineering. Biocompatibility, biodegradability, and osteoconductivity are the inherent features of chitosan for bone tissue engineering; moreover, chitosan's multiple active amine groups allow for easy chemical modifications of the biomaterial according to the specific type of defects.^25^ In cartilage repair, however, the performance of chitosan alone is more limited. Chitosan-based scaffolds on their own are a reasonable choice for articular cartilage regeneration but prove insufficient for the regeneration of more structurally complex or mechanically demanding cartilage types, and alginate-chitosan polyelectrolyte complex hydrogels, despite producing some cartilage at injury sites, did not generate a significant regenerative response on their own without the addition of therapeutics or growth factors. This finding is important for translational design: chitosan alone cannot bridge the gap from laboratory performance to clinical adequacy in load-bearing applications, and its combination with ceramic nanofillers or synthetic polymers is therefore not optional but necessary. Beyond mechanical limitations, the clinical utility of chitosan is also impeded by inadequate haemocompatibility, insufficient durability under physiological loading, and a lack of uniformity in commercial manufacturing obstacles that must be resolved before chitosan-based scaffolds can be reliably scaled for widespread clinical use.^26^

Alginate is a natural polysaccharide obtained from brown seaweed and marine algae. Structurally, it consists of two uronic acid monomers β-d-mannuronic acid and α-l-guluronic acid connected by 1,4-glycosidic bonds.^27^ One of alginate's most practical advantages is its ability to form a stable hydrogel very quickly when it comes into contact with divalent cations such as calcium ions, which means it can be injected into irregularly shaped defects and conform precisely to their geometry; a feature that is difficult to achieve with prefabricated solid scaffolds. Ionic crosslinking interactions by which alginate can be crosslinked by divalent cations such as Ca^2+^ have been particularly widely used to prepare hydrogels for tissue engineering, given the high cell compatibility of these crosslinkers; however, ion exchange between the crosslinking cations and physiological ions in the body can make it difficult to control the degradation behaviour of alginate hydrogels *in vivo*.^28^ The fundamental translational limitation of alginate is its mechanical weakness: under the compressive and shear loading conditions present in most clinically relevant tissue sites particularly bone and cartilage pure alginate hydrogels deform and fail. This problem has not been satisfactorily solved by gelation chemistry alone, and reinforcement with ceramic nanoparticles or fibre networks is required for load-bearing applications.^29,30^

##### 2.1.1.2 Protein-based polymers

Collagen is the most abundant protein in the human body, forming the primary structural component of the ECM in virtually all connective tissues including bone, cartilage, tendon, skin, and blood vessels. It is composed of three polypeptides arranged in a unique triple helix tropocollagen structure to form fibers and fibrils that serve as a mechanical foundation for the different types of tissues.^31^ Collagen's success rate is unmatched among all natural polymers in the field of tissue engineering due to the nature of its biological activity and the fact that it is an immunologically inert material and thus, the safest choice when developing scaffolds intended to replicate the extracellular matrix.^32^ Nevertheless, there are several shortcomings associated with the clinical application of collagen scaffolds. First, their mechanical strength is insufficient for applications involving loads. Second, due to the presence of epitopes, it could cause immunogenic responses in the recipients. Third, fast enzymatic degradation makes the collagen material unstable before complete differentiation of target tissues. For these reasons, collagen scaffolds are only used in applications requiring minimal strength such as wound dressing. Enhancing collagen scaffold stability through chemical or physical crosslinking can prolong bioactivity and mechanical performance, but crosslinking at standard concentrations can also negatively impact integration with native tissue by blocking cell-binding sites, and strategies that minimise crosslinker concentration while maintaining structural integrity remain an active area of research. Critically, the most promising recent direction for collagen in high-demand applications is its incorporation into nanocomposite systems combined with hydroxyapatite for bone, with carbon nanofibers for vascular grafts, or with synthetic polymers for load-bearing cartilage rather than its use as a standalone scaffold.^33,34^

Gelatin is produced by the partial hydrolysis of collagen under heat and acidic or alkaline conditions, which disrupts the triple helix while preserving the backbone amino acid sequence. Because gelatin retains the same arginine-glycine-aspartate (RGD) cell-adhesion sequences as collagen, it is inherently cell-adhesive without requiring surface functionalisation.^35^ Gelatin scaffolds contain RGD binding motifs for cell adhesion and degrade into non-toxic resorbable products; however, the low mechanical stability of gelatin hydrogels at physiological temperatures in the absence of chemical crosslinks typically imparts poor mechanical strength, meaning gelatin scaffolds cannot maintain structural integrity under the loading conditions present in most tissue engineering applications without reinforcement.^36,37^ Both chitosan and gelatin present limitations common to natural polymers reduced mechanical strength and high degradation rate and these limitations are addressed by crosslinking, which involves forming bonds between polymer chains to improve mechanical and degradation properties; crosslinking is therefore an essential step in the fabrication of functional natural polymer-based scaffolds. Gelatin methacryloyl (GelMA), produced by reacting gelatin with methacrylic anhydride to introduce photopolymerisable groups, has emerged as the most widely used gelatin-based bioink for 3D bioprinting precisely because UV crosslinking allows its mechanical properties to be tuned on demand. GelMA-based nanocomposites incorporating carbon nanotubes, hydroxyapatite, or clay nanoplatelets represent one of the most actively researched scaffold platforms currently approaching clinical evaluation.^38,39^

#### 2.1.2 Synthetic polymers

Synthetic polymers are produced through controlled polymerisation of organic monomers, and their properties including degradation rate, mechanical stiffness, hydrophilicity, and porosity can be independently adjusted during the synthesis. This predictability and reproducibility give them a decisive practical advantage over natural polymers for clinical translation: every batch can be made to the same specification, which is a prerequisite for regulatory approval. Biodegradable synthetic polymers such as PLA, PGA, PCL, and PLGA are widely used in tissue engineering and clinical settings because of their excellent biocompatibility, controlled degradability, and mechanical strength; PLA is thermally stable and decomposes into non-toxic by-products, PLGA is FDA-approved for its controlled degradability and biocompatibility, and PCL is another FDA-approved polymer used in bone repair that features a low melting point, good miscibility, and excellent blend compatibility with other polymers. However, synthetic polymers are not without meaningful drawbacks, and a critical understanding of when each system succeeds or fails is essential for rational scaffold design.^40,41^

Polycaprolactone (PCL) is produced by ring-opening polymerisation of ε-caprolactone and degrades slowly over 2–4 years in the body by hydrolytic cleavage. This slow degradation makes it well suited for long-term structural applications such as bone scaffolds and vascular grafts, but unsuitable for applications requiring faster tissue remodelling, such as wound healing or neural conduits.^42,43^ Its highly hydrophobic surface water contact angle of 80–90° reduces protein adsorption and cell adhesion compared to natural polymers, which is why PCL is rarely used alone and is instead blended with collagen, gelatin, or ceramic nanoparticles to improve its biological performance. The combination of PCL with PLGA is one of the most frequently reported systems for cartilage repair precisely because each polymer compensates for the other's weakness: a PCL/PLGA (80 : 20) scaffold showed appropriate physicochemical properties confirmed by FTIR and SEM, demonstrated non-toxic effects, supported dental follicle mesenchymal stem cell adhesion and glycosaminoglycan synthesis *in vitro*, and showed better repair capacity than two commercial comparators in a rat chondral defect model highlighting that the blend's combined properties outperform either polymer alone.^44,45^

Polylactic acid (PLA) is synthesized using plant-derived lactic acid, has the ability to produce two optical forms, PLLA and PDLA, with their percentage controlling the extent of crystallinity and the rate of degradation. PLA is biocompatible with cells but has a non-polar surface that repels cell adhesion; its breakdown products, including lactic acid, induce local acidosis and trigger mild inflammation. However, the degradation process of PLLA, which takes between 12 to 24 months, suits bone regeneration but is unsuitable for soft tissue repair.^46–48^

Polyglycolic acid (PGA) is very similar in structure to polylactic acid (PLA), but there is no methyl side chain, giving it increased water solubility and faster degradation. This leads to quick production of acidic degradation products and quick reduction of mechanical strength; hence, it can only be used for temporary scaffolds or small amounts in composite materials.^49^

Poly(lactic-*co*-glycolic acid) (PLGA) addresses the inherent drawbacks of PLA and PGA through copolymerization, enabling control over degradation speed along the entire spectrum from PLA to PGA based on the ratio of the monomers used. PLGA has the greatest clinical record of all synthetic polymers in tissue engineering applications, and the FDA approval for the use of PLGA in medical devices grants it a distinct regulatory advantage over any newly developed polymer. The main drawback with PLGA is still associated with the high cost of raw materials and controlled synthesis techniques compared to PCL or PLA.^50,51^

Poly(*N*-isopropylacrylamide) (PNIPAAm) is a thermoresponsive polymer that undergoes a reversible collapse at its lower critical solution temperature (LCST) of approximately 32 °C, shifting from a swollen, water-absorbing state at room temperature to a contracted state at body temperature.^52^ This property enables injectable scaffold applications where the material is delivered as a liquid and gels *in situ*. However, PNIPAAm in its pure form is not biodegradable, which remains a fundamental barrier to clinical translation, and it is therefore used only as a minor functional component within biodegradable polymer blends.^53^

### 2.2 Based on reinforcement type

The addition of nanoscale reinforcing phases into polymer matrices is what distinguishes nanocomposites from conventional polymer blends and is the principal reason for their superior tissue engineering performance. The geometry and chemistry of the filler whether particles, fibres, or tubes determines which properties are enhanced and for which tissue types the resulting scaffold is best suited.

#### 2.2.1 Carbon nanotubes (CNTs)

Carbon nanotubes are hollow cylindrical structures formed by rolling one or more layers of graphene, with walls composed of sp^2^-hybridised carbon atoms in a hexagonal arrangement. They are classified into single-walled (SWCNTs), double-walled (DWCNTs), and multi-walled (MWCNTs) types based on the number of concentric graphene cylinders. CNTs and their polymer composites exhibit considerable potential for tissue engineering because of their unique combination of mechanical strength, electrical conductivity, chemical modifiability, and antimicrobial activity properties that together make them ideal candidates for constructing biological scaffolds that support tissue regeneration and enhance cellular function across bone, neural, and cardiac applications.^54^ In neural tissue engineering, CNT scaffolds provide the electrical conductivity required to mimic the electroactive microenvironment of native neural tissue, which conventional polymer scaffolds cannot replicate. In cardiac tissue engineering, aligned CNT networks have been used to guide cardiomyocyte orientation and improve the formation of gap junctions, improving the electrical coupling between cells that is essential for synchronised contraction. Electrospun PCL/gelatin/CNT yarns fabricated into fabric-like scaffolds showed good biocompatibility, guided cell elongation, and produced mechanical properties similar to native blood vessels suggesting practical potential in vascular tissue regeneration as well.^55,56^ However, CNTs carry a well-documented translational problem: cytotoxicity. Cytotoxicity remains a significant concern for CNTs, potentially linked to factors including CNT concentration, diameter, length, and surface chemistry, and research into functionalization strategies particularly carboxylation and hydroxylation and composite formation to mitigate toxicity while maintaining performance is an active area of investigation.^57^ Studies have focused on incorporating optimal CNT concentrations that limit cytotoxicity while maintaining mechanical and electrical performance improvements; both approximately 2% and approximately 5% CNT loadings in electrospun poly(*p*-dioxanone)/CNT nanoyarns exhibited no cytotoxic effects and provided a nanofibrous topography with high surface area that regulated cell morphology through contact guidance, though higher CNT concentrations negatively affected electrospinning processability. This dose-dependence of CNT toxicity means that scaffold design requires careful optimisation of filler concentration a step that is frequently underreported in the literature and that constitutes a genuine barrier to standardised scale-up and regulatory submission for CNT-containing devices. The long-term biocompatibility of CNT-containing scaffolds particularly concerning potential immune responses, cytotoxicity, and degradation behaviour requires extensive *in vivo* validation before clinical translation can be realised, and this gap between promising *in vitro* results and the evidence required by regulatory agencies represents the principal bottleneck for CNT-based tissue engineering systems.^58,59^

#### 2.2.2 Carbon nanofibers (CNFs)

Carbon nanofibers are solid or hollow cylindrical carbon structures with diameters of 10–100 nanometres, which distinguishes them structurally from the hollow-walled CNTs.^60^ They are produced primarily by catalytic chemical vapour deposition or by electrospinning a polymer precursor (typically polyacrylonitrile) followed by carbonisation at high temperature. CNFs possess excellent inherent and tunable physical, chemical, mechanical, electrical, thermal, and optical properties, and their high surface-area-to-volume ratio combined with a three-dimensional fibrous architecture closely resembling the collagen fibrils of the native ECM makes them particularly well suited for scaffolds designed to guide cell adhesion, growth, proliferation, and differentiation into specific tissue types.^61,62^ Compared with CNTs, CNFs offer a practical advantage for translational applications: their surface chemistry is simpler to modify, their cytotoxicity profile under physiological conditions is generally lower, and the more straightforward fabrication route allows greater batch-to-batch consistency. CNF incorporation into polymer scaffolds has demonstrated osteogenic, chondrogenic, and neural differentiation-promoting effects *in vitro*, and CNF-reinforced PCL scaffolds have shown promising *in vivo* bone regeneration performance in small animal models without adverse inflammatory responses at 28 days.^63^

For both CNTs and CNFs, the same fundamental gap applies: there is no standardised *in vitro-in vivo* correlation model that allows preclinical results from small animals to predict clinical performance reliably. The absence of agreed threshold values for acceptable nanofiller systemic exposure following scaffold degradation *in vivo*; a regulatory gap explicitly identified by both the FDA and EMA; means that every new nanofiller-containing scaffold system must undergo the full safety characterisation sequence independently, at considerable cost and time. Until this gap is closed through coordinated regulatory guidance and the development of validated surrogate models, the clinical translation of CNT and CNF-reinforced nanocomposite scaffolds will remain slower than their laboratory performance justifies.

## 3. Synthesis and fabrication techniques of polymeric nanocomposites

**Fig. 3:**
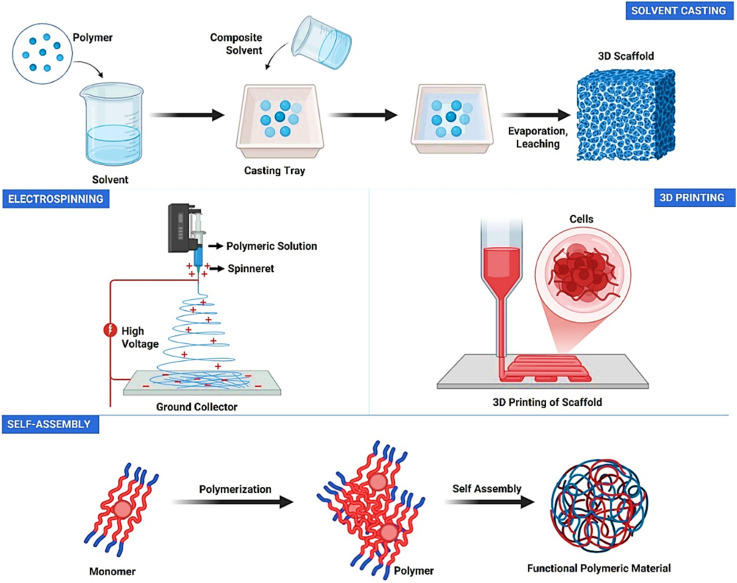
Scaffold fabrication technologies. The images were created with Biorender.com.

The choice of a fabrication technique is one of the primary decisions made during the design phase of a scaffold, which affects its architecture and material–cell interaction and thereby the ability to translate the construct into clinical applications. There are four main techniques, including solvent casting, electrospinning, 3D printing, and self-assembly. All four techniques offer unique advantages and limitations, and none can be used interchangeably. Each of the techniques is outlined below. Overview of key scaffold fabrication techniques used in tissue engineering and drug delivery applications is shown in Fig. 3. Comparative overview of major scaffold fabrication techniques used in tissue engineering is given in Table 1.

**Table 1:** Comparative analysis of scaffold fabrication techniques for tissue engineering

Parameter	Solvent casting	Electrospinning	3D printing	Self-assembly
Pore size	100–500 µm	0.1–5 µm	100–600 µm	5–200 nm
Mechanical strength	Low-moderate	Low-moderate	Moderate-high	Very low
Cell viability	Post-fab seeding	Post-fab seeding	High (bioprinting)	High
Scalability	Easy	Moderate	Moderate-difficult	Difficult
Regulatory status	Established precedent	Established precedent	Evolving (FDA 2023)	Preclinical mainly
Best tissue application	Bone, cartilage	Skin, nerve, vascular	Bone, cartilage, vascular	Nerve, cartilage
Key limitation	Residual solvent toxicity; limited pore control	Limited scaffold thickness; poor 3D cell infiltration	High equipment cost; regulatory novelty	Weak mechanics; poor scalability; slow gelation

### 3.1 Solvent casting

One of the most popular and versatile methods of scaffold preparation based on the use of polymers is solvent casting, recognized for its relative ease, inexpensive equipment, and suitability for use with a large variety of synthetic and natural polymers. As per this method, the selected polymer is initially dissolved in an organic solvent to produce a homogeneous polymer solution.^64^ After that, porogens, typically sodium chloride, paraffin spheres, or sugar crystals of predetermined sizes are distributed in a polymer solution, which is poured into a mold with the intended geometrical configuration. Finally, once the solvent evaporates, the porogens are leached out using a selective solvent or distilled water, resulting in the formation of a scaffold with pores of certain diameter. Solvent casting is capable of producing scaffolds with controllable porosity since porogen sizes determine pore sizes. But at the same time, controlling the pore shape and interconnectivity becomes problematic, as such parameters depend on the porogen properties.^65^

The major drawback associated with this method for clinical applications is solvent toxicity. Scaffolds prepared using solvent casting and particulate leaching processes can contain organic solvents, which will have adverse effects on mechanical properties, biocompatibility, and permeability. These factors create problems in securing approval from relevant authorities like the FDA. The elimination of solvents or reduction in their concentration through vacuum drying and ICH Q3C compliance is therefore critical for all clinically applicable scaffolds prepared by solvent casting processes. This issue is not only related to the preparation process but also has regulatory importance. Dichloromethane and chloroform, which are widely used to dissolve PCL and PLGA, belong to the category of ICH Class 2 solvent with defined daily exposure restrictions. Solvent-free alternative approaches including melt moulding, gas foaming, selective laser sintering, and supercritical CO_2_ foaming avoid the regulatory complexity of residual solvent management, but they require post-processing pore-generation steps such as particulate leaching or phase separation to achieve the scaffold porosity required for tissue engineering; this means that solvent-free approaches gain regulatory simplicity at the cost of fabrication complexity.^66^

Despite these limitations, solvent casting retains important practical advantages. It requires no specialist equipment, is compatible with bioactive agent incorporation post-fabrication, and produces scaffolds with straightforward and well-characterised geometry. For bone and cartilage applications where, complex architectures are not required and cost is a primary constraint, solvent casting combined with particulate leaching remains a clinically relevant and regulatory-precedented approach. A direct comparison of electrospun and solvent-cast PCL-PLGA blends demonstrated that solvent-cast scaffolds showed a characteristic bubble-like surface topography derived from the spherical porogen particles, with lower water contact angles (∼74°) compared with electrospun counterparts (∼110°), meaning greater hydrophilicity and, in consequence, improved cell adhesion confirming that solvent casting can produce biologically favourable surface characteristics that compensate in part for its architectural limitations.^67^

### 3.2 Electrospinning

Electrospinning is the most widely used technique for producing nanofibrous scaffolds in tissue engineering, and its dominance reflects a genuine and well-evidenced advantage: the ability to produce fibres with diameters from 10 nm to several micrometres that structurally resemble the collagen fibril network of the native ECM at a scale that no other fabrication method routinely achieves. In the electrospinning process, a polymer solution held in a syringe under high voltage develops a conical deformation at the tip, from which a charged polymer jet is ejected toward a grounded collector. As the solvent evaporates during transit, the polymer solidifies into ultrafine fibres deposited as a nonwoven mesh. The high surface-area-to-volume ratio of electrospun nanofibres, combined with their structural ability to mimic the ECM at the nanoscale, facilitates cell migration, proliferation, adhesion, and differentiation properties that make electrospinning the most practically relevant scaffold fabrication method for thin, membrane-type tissue engineering applications including skin, peripheral nerve guidance conduits, and vascular grafts. The direction of fibre alignment is an important and controllable architectural variable.^68^ Random fibre networks produce isotropic scaffolds suitable for skin and bone applications; aligned fibre networks provide topographical contact guidance cues that direct cell elongation along the fibre axis, making them particularly valuable for nerve and tendon repair where cellular and ECM anisotropy is a functional requirement. The pore size of the electrospun scaffold is essential for supporting cell–cell and cell–matrix interactions, with the optimal pore size being cell-type dependent; the stiffness of the electrospun ECM analogue also directly influences cellular activities including adhesion, migration, proliferation, and differentiation; meaning that both the fibre architecture and the mechanical properties of the network must be designed simultaneously rather than independently.^69,70^

However, conventional electrospinning has two persistent limitations that fundamentally restrict its clinical application. First, traditional electrospun membranes produced by single-stage spinning are thin typically less than 1 mm and have closely packed fibre arrays that create pores too small for efficient three-dimensional cell infiltration. Traditional two-dimensional electrospun membranes have an intrinsic limitation of relatively poor cellular infiltration due to their limited thickness and high fibre packing density, which causes them to function as two-dimensional surfaces rather than genuine three-dimensional microenvironments; three-dimensional electrospun nanofibre-based scaffolds that provide stem cells with genuine 3D microenvironments and biomimetic fibrous structures have been developed to address this limitation and have demonstrated improved tissue repair outcomes across multiple tissue types. Second, electrospinning relies on organic solvents for most synthetic polymer systems, carrying the same residual solvent management challenges as solvent casting. Although electrospinning's biocompatibility, tunable fibre architecture, and ease of surface functionalisation are well-established advantages, challenges including the toxicity of organic solvents and limitations in scalability persist, limiting clinical translation; emerging approaches including green solvent electrospinning and integration with 3D printing and microfluidics represent the most promising directions for overcoming these practical barriers.^71–73^

The recent development of combination of electrospinning with 3D bioprinting to produce hierarchical scaffolds that simultaneously offer nanoscale ECM mimicry (from electrospun fibre layers) and macroscale-controlled architecture (from 3D-printed framework elements). This hybrid approach directly addresses the thickness and cell infiltration limitations of electrospinning alone, and represents one of the most active current frontiers in scaffold fabrication research.^74,75^

### 3.3 3D printing

Three-dimensional printing has transformed tissue engineering by enabling the fabrication of patient-specific, architecturally precise scaffolds from digital design files a capability that no other fabrication method can match.^76^ The defining principle is computer-aided design (CAD)-guided layer-by-layer deposition of material, which allows precise control over pore geometry, strut dimensions, porosity, and external shape simultaneously. 3D bioprinting methods including inkjet, extrusion-based, laser-assisted, and digital light processing (DLP) approaches can fabricate complex, multi-material structures with high precision in geometry, material composition, and cellular microenvironments, enabling the incorporation of biomimetic design principles that replicate both the mechanical and biological behaviours of native tissues.^77^

3D printing encompasses two fundamentally different strategies: scaffold-based and scaffold-free bioprinting. Scaffold-based printing uses synthetic polymers, hydrogels, ceramics, or their composites to create defined structural templates that support cell colonisation and tissue formation. Scaffold-free printing uses only dense cellular aggregates such as spheroids or cylindrical cell rods without foreign scaffold material, relying on cell–cell adhesion and self-organisation to produce tissue-like constructs. While scaffold-free approaches offer the advantage of eliminating potentially immunogenic scaffold materials, they provide no immediate structural support and are therefore limited to applications where the target tissue is itself mechanically soft and does not require load-bearing capacity.^78^

The four major 3D bioprinting modalities each carry a distinct performance trade-off that directly determines their application.^79^

Extrusion-based bioprinting deposits cell-laden or acellular bioinks through pressure- or temperature-controlled nozzles, accommodating a wide range of bioink viscosities from low-viscosity hydrogels to high-viscosity polymer melts; cell viability ranges from 40–80% and spatial resolution is approximately 200 µm adequate for most tissue engineering architectures but insufficient for replicating capillary-scale vascular structures. Inkjet bioprinting ejects precise droplets of low-viscosity bioink under thermal or piezoelectric impulse, achieving cell viability of approximately 90%, fast printing speeds, and high resolution at approximately 20–100 µm, but is limited to low-viscosity materials and is prone to clogging with cell-laden bioinks. Laser-assisted bioprinting transfers bioink droplets using laser energy without a nozzle, achieving cell viabilities exceeding 95% and spatial resolution below 20 µm; the best performance of any technique but at high equipment cost and with risk of metallic contamination from the laser-absorbing layer. Stereolithography-based bioprinting photopolymerises resin-based bioinks layer by layer, achieving the highest fabrication accuracy at resolutions of 1.2–200 µm with fast build speeds, but is limited to photopolymerisable materials and UV light exposure can reduce cell viability at extended print durations.^80,81^

3D printing carries a decisive practical advantage for clinical translation that is not always made explicit in the literature: its inherent digital reproducibility. Because the same CAD file produces nominally identical scaffolds across batches from the same machine, lot-to-lot consistency can in principle be mathematically validated rather than empirically demonstrated for each batch; a GMP compliance advantage over the inherently more variable solvent casting and electrospinning methods. 3D bioprinting has been applied to fabricate human-sized ear cartilage constructs that maintain shape and support cartilage tissue formation after implantation, as well as tubular vascular constructs showing cell proliferation and matrix remodelling *in vitro*, demonstrating that the technology can produce clinically relevant tissue volumes and geometries though translational studies confirming *in vivo* performance at human scale remain the critical evidence gap.^82^

### 3.4 Self Assembly

Self-assembly is a bottom-up fabrication strategy in which molecules spontaneously organise into ordered nanostructures through non-covalent interactions such as hydrogen bonding, hydrophobic forces, electrostatic interactions, van der Waals forces, and π–π stacking without any external templating or mechanical input.^83^ In tissue engineering, self-assembling peptide (SAP) hydrogels are the most developed class of self-assembled scaffold, formed from short amino acid sequences designed to fold into β-sheet or α-helix secondary structures that then associate laterally into nanofibres and macroscopic hydrogel networks. Self-assembling peptides offer a promising alternative to conventional hydrogels because they form micro- and nanostructured hydrogels through non-covalent interactions, allow precise control over biofunctionality and mechanical properties through rational sequence design, and can dynamically regulate assembly and degradation in response to specific stimuli including pH, ionic strength, enzymatic cleavage, and temperature; a combination of properties that is difficult to achieve with conventional polymer hydrogels.^84^

The pore architecture of SAP hydrogels typically 5–200 nm is too fine to permit active cell migration into the network in the absence of enzymatic or hydrolytic remodelling. Cell colonisation therefore depends on local scaffold remodelling by the cells themselves, a process that is relatively slow and spatially constrained compared with the macroporosity of solvent-cast or 3D-printed scaffolds. The mechanical properties of SAP hydrogels are highly sensitive to amino acid sequence, peptide concentration, pH, temperature, and electrolyte composition; parameters that are straightforward to control in the laboratory but difficult to maintain constant during clinical-scale manufacturing and product shelf-life management. For clinical use, SAP scaffolds must satisfy demanding specifications simultaneously: biomechanical properties matching the target tissue, multiple functional motifs for cell and cytokine interaction, a bioabsorption time compatible with tissue regeneration kinetics, and nanostructure and porosity facilitating tissue infiltration and cytokine diffusion; a combination of requirements that has limited the clinical translation of SAP scaffolds to relatively soft, low-mechanical-demand applications including corneal repair, cardiac tissue support, and neural regeneration despite their excellent laboratory performance.^85,86^

The most important recent advance in self-assembling scaffold technology is the integration of SAP hydrogels with extrusion-based 3D bioprinting. The self-assembled peptide hydrogel scaffold has nanofibrillar structure, tunable mechanical properties, and suitable biodegradability that enable the formation, growth, and controlled release of cell spheroids with uniform size for 3D bioprinting, tissue engineering, and organ regeneration; mammary epithelial cells encapsulated in 3D-printed SAP hydrogel matrices remained viable and began proliferating after seven days in culture independent of hydrogel stiffness demonstrating that SAP hydrogels can function as printable bioinks that combine structural definition from the printer with ECM-mimicking nano-architecture from self-assembly.^87,88^

In light of the comparative analysis outlined above, the choice of method becomes clear. For devices whose structure necessitates patient-specific complex geometry, 3D printing is the only option available. Electrospinning should be used for creating membrane-like structures because of its better fiber alignment and ECM simulation capabilities. For devices that are meant to be injected into patients, self-assembling hydrogels are recommended even though their mechanics are inferior. Solvent casting will remain relevant when simpler geometries are needed.

## 4. Characterization of polymeric nanocomposites

### 4.1 Mechanical properties

The fundamental mechanical design principle in tissue engineering is mechano-compatibility: a scaffold must match, or at least not dramatically depart from, the mechanical properties of the tissue it is intended to replace or support. Human tissues span a mechanical range from millipascals to gigapascals, and they exhibit non-linear viscoelasticity with strain-stiffening behaviour; the design challenge for biopolymer composites is therefore not simply achieving a target stiffness value but replicating the non-linear mechanical response of living tissue across the full range of physiological loading conditions.^89^ A scaffold that is too stiff will cause stress-shielding and bone resorption; one that is too compliant will fail mechanically before tissue regeneration is complete. This dual constraint mechanical sufficiency and tissue compatibility; is why no single polymer can meet the requirements across different tissue types, and why nanocomposite reinforcement is the only practical engineering strategy.^90^ The mechanical advantage of nanoparticle reinforcement over conventional microparticle additives arises directly from the surface-area-to-volume relationship of the nanoscale. Carbon-based nanostructures including graphene oxide, reduced graphene oxide, carbon nanotubes, fibrous carbon nanostructures, and nanodiamonds substantially improve the mechanical performance of polymer matrices; their tubular or planar structure allows them to transfer external loads from the outer polymer matrix to the inner layers of the composite, and they simultaneously create porosity above 80% necessary for tissue engineering by their spatial arrangement in the scaffold architecture. Silver (Ag) nanoparticles are among the most extensively studied metal nanoparticle reinforcers. Their incorporation into polyurethane (PU) matrices increases tensile strength, impact resistance, and chain storage modulus without compromising material flexibility a combination that is difficult to achieve with larger particulate additives. Similarly, Ag nanoparticles improve the compressive strength and durability of chitosan/polyvinyl alcohol (PVA) composite matrices, making them suitable for both load-bearing and wound-healing applications. The mechanistic basis for these improvements is well-established: the high modulus of the nanoparticle, its large surface area, and the intimate interfacial contact between particle and polymer chain restrict chain mobility under stress, increasing resistance to deformation. Carbon-reinforced PEEK composites represent a recent high-performance direction for hard tissue applications. Carbon fibre-reinforced PEEK (CFR-PEEK) composites fabricated by fused deposition modelling improved tensile, bending, and compressive strength substantially compared with pure PEEK, with biological tests confirming good biocompatibility demonstrating that carbon fibre reinforcement can bring polymer-based composites into the mechanical range required for dental and orthopaedic applications while retaining the processability advantages of polymer-based systems. PLA/Fe_3_O_4_ nanocomposite scaffolds with a gyroid porous structure represent a critical recent example of how nanoparticle addition can tune mechanical properties to match a specific tissue: 3D-printed PLA/Fe_3_O_4_ composite scaffolds with 50% porosity produced mechanical properties such as Young's modulus, compressive strength, and tensile strength that fell within the range of corresponding properties of native cancellous bone, confirming that the combination of nanoparticle addition and controlled porosity design allows mechanical properties to be targeted to specific bone types rather than broadly approximated. This demonstrates the significance of the design approach, which states that reinforcement at the nanocomposite level should be used alongside architectural design for achieving desired mechanical behavior; neither can succeed on its own. Table 2 provides an overview of the mechanical properties of various polymeric nanocomposite materials. A critical reading of this data reveals three important patterns. First, PCL-based composites occupy the widest useful mechanical range across tissue types, from the low moduli required for vascular grafts (1.93 MPa Young's modulus) to the moderate stiffness needed for bone scaffolds when reinforced with HA (∼100 MPa elastic modulus). Second, PLGA systems offer intermediate mechanical performance (Young's modulus ∼48.6 MPa) that is well-suited to rotator cuff and soft orthopaedic applications, but their acidic degradation products can progressively alter the local mechanical environment as the scaffold breaks down a problem that does not appear in the mechanical testing data but is critical for long-term *in vivo* performance. Third, PVDF while offering adjustable fibre architecture through electrospinning (fibre diameter 0.2–0.8 µm) provides electrical stimulation capability but does not offer the mechanical compliance required for most soft tissue applications without composite modification.^91,92^

**Table 2:** Mechanical properties of representative polymeric nanocomposite systems for tissue engineering

Polymer system	Mechanical property	Value	Target tissue	Key limitation	Ref.
PCL (electrospun)	Tensile strength	6.8 ± 0.81 MPa	Oesophageal scaffold	Hydrophobic surface limits cell adhesion	93
Chitosan/PVA	Tensile strength	6.15 MPa	Soft tissue engineering	Low modulus for load-bearing sites	94
PCL/collagen composite	Tensile strength	2.02 MPa	Vascular/soft tissue	Rapid collagen degradation *in vivo*	95
Collagen/glutaraldehyde	Tensile strength	6.72 ± 0.44 MPa	Artificial skin	GTA cytotoxicity at high concentrations	96
PCL/chitosan composite	Elastic modulus	7.8 ± 0.5 MPa	Liver tissue engineering	Insufficient stiffness for hepatic ECM mimicry	97
PCL/hydroxyapatite	Elastic modulus	∼100 MPa	Bone scaffolds	Brittleness under cyclic fatigue loading	98
PCL (3D-printed)	Elastic modulus	6.7 ± 0.4 MPa	Dynamic load-bearing soft tissue	Slow degradation rate (2–4 years)	99
PCL (electrospun)	Young's modulus	1.93 ± 0.36 MPa	Vascular tissue engineering	Insufficient endothelialisation without surface functionalisation	99
PLGA	Young's modulus	48.6 MPa	Rotator cuff/soft orthopaedic	Acidic degradation products provoke local inflammation	100
PVDF (electrospun)	Fibre diameter	0.2–0.8 µm	Piezoelectric/electrostimulation scaffolds	Non-biodegradable; regulatory uncertainty	101

### 4.2 Morphology and surface characterization

The morphology of a polymeric nanocomposite scaffold; its pore size, fibre diameter, surface roughness, and nanoscale topographical features is not merely descriptive. It is mechanistically linked to cell adhesion, migration, proliferation, and lineage commitment, because cells sense and respond to the physical geometry of their substrate through integrin-mediated mechanotransduction pathways. The development of nanostructured surfaces of polymeric nanocomposites has garnered increasing attention in tissue engineering and regenerative medicine because nanotopographical features at sub-micron and nanometre scales significantly influence the physical, chemical, and biological properties of biomaterials, affecting both their interactions with individual cells and the surrounding tissue response at the implant level. Transmission electron microscopy (TEM) is the primary tool for characterising the internal dispersion state of nanofillers within polymer matrices; a characterisation need that has no equivalent at lower magnification. For one-dimensional fillers (CNTs), TEM reveals the degree of bundling, alignment, and debundling that determines whether load transfer from polymer to filler actually occurs. For two-dimensional fillers (graphene oxide, clay nanoplatelets), TEM reveals whether exfoliation into individual sheets has been achieved; a critical determinant of the surface-area-to-volume ratio available for polymer–filler interaction. For three-dimensional nanoparticles (HAp, ZnO, Ag), TEM provides direct measurement of particle diameter, agglomeration state, and interfacial bonding quality. These parameters cannot be inferred from bulk mechanical testing alone and must be characterised by TEM to validate the dispersion assumptions underlying composite design.^102,103^

Scanning electron microscopy (SEM) provides complementary surface morphology data at a length scale directly relevant to cell–scaffold interactions, typically imaging pore geometry, interconnectivity, fibre architecture, and surface texture. SEM imaging, in conjunction with environmental SEM (ESEM) and cryo-SEM techniques, allows characterisation of hydrogel microarchitecture including pore size distribution and wall thickness, and these parameters are directly linked to the scaffold's ability to support nutrient transport, cell infiltration, and waste removal; all of which are rate-limiting for tissue regeneration in three-dimensional constructs. Surface roughness measured from SEM analysis is functionally significant: surfaces with mean roughness (Ra) values of approximately 0.5–2 µm have been consistently shown to promote osteoblast adhesion compared with smoother or rougher surfaces, and this roughness dependence is one mechanism by which nanoparticle incorporation which naturally increases surface texture improves cell attachment even without chemical surface functionalisation.^104^ In biocompatibility assessment, SEM plays a critical quality control role: SEM images of PCL/PLA/ZnO nanoparticle composite scaffolds showed that increasing ZnO-NP content produced a more compact and homogeneous surface due to excellent nanoparticle dispersion and accumulation between polymer chains confirming that SEM can directly reveal the quality of nanoparticle incorporation and predict the resulting biological behaviour without requiring cell culture experiments.^105^

AFM offers capabilities beyond those of SEM for nanocomposite scaffold characterisation: it can quantify surface roughness at nanometre resolution, map local elastic modulus across a heterogeneous composite surface through force–distance curve analysis, and discriminate between phases of different stiffness in the same material using phase contrast imaging without chemical staining. AFM is currently considered the best tool for surface characterisation of polymers and biomaterials, outputting high-resolution images of micro- and nanotextured scaffold surfaces; by capitalising on atomic force spectroscopy and tip-sample local interactions, it allows simultaneous collection of elasticity, viscoelasticity, surface charge density, and wettability data; information that is collectively required to predict cell–scaffold interaction behaviour. Phase imaging mode is particularly valuable for multi-component nanocomposites, where it allows mapping of the spatial distribution of hard nanoparticle and soft polymer domains without destroying the sample, enabling direct correlation of filler distribution with local mechanical properties.^106,107^

### 4.3 Biocompatibility and biodegradability testing

Biocompatibility is a property of a material that allows it to fulfil its purpose without eliciting a hostile body response. Biocompatible polymers have received massive respect in recent decades owing to their various applications in tissue engineering, treatment of genetic disorders, and drug delivery. Biocompatibility testing is a critical requirement for the regulatory approval of polymer-based medical devices.^108^*In vitro* studies assess cytotoxicity, cell adhesion, proliferation, and differentiation using standard methodologies such as metabolic activity assays, morphological evaluation, and live-cell staining. For example, Chitosan/graphene oxide (GO) scaffolds have been found to be more effective in enhancing preosteoblast proliferation and metabolic activity in bone tissue engineering. The microscopy findings of the stained samples using the phase-contrast method clearly showed clear pores and increased cell attachment, which represents the great bioactivity of the scaffold. Similarly, osteosarcoma cells proliferated and attached to electrospun hydroxyethyl cellulose (HEC)/polyvinyl alcohol (PVA) nanofibrous scaffolds. They were porous, with their porosity emulating the extracellular matrix, which enhanced the diffusion of nutrients and cell migration. Strong cell adhesion and growth for 10 days were destroyed by scanning electron microscopy and MTS assays. A different experiment on carbon nanofiber (CNF)-reinforced 3D poly-ε-caprolactone (PCL) scaffolds revealed that the structures enhanced the development of meniscal cells and their extracellular matrix. Hoechst staining and MTT assays confirmed high cell viability and integration within the scaffolds.^109^*In vivo* studies assess material safety and functionality under physiological conditions. An example is a CNF-reinforced PCL scaffold implanted under the skin of rabbits to determine the biocompatibility of the scaffold. No inflammatory response or systemic toxicity was observed after 28 days.^110^ Histological analysis showed successful tissue integration and cell proliferation. Another analysis involved the use of a scaffold made of 2% poly(3-hydroxybutyrate-*co*-3-hydroxyvalerate) (PHBV) reinforced with multi-walled carbon nanotubes (MWCNTs) implanted in bone defects in rats. The histological study and micro-computed tomography (micro-CT) showed that the osteogenesis and bone volume were improved, provided that the polymer could degrade in the human body after six weeks; that is, the bone integration and bone healing were improved.^108^ Biodegradability testing is based on analyzing whether the polymer was able to degrade in the human body, that is, whether the rate at which the polymer degraded matched the rate at which new tissue grew. Considering the difference in the healing process of 10 to 18 months based on the tissue type and extent of damage, biodegradable scaffolds should be strong enough to hold the place when the tissue is healing and be able to break down at a suitable rate. Polylactic acid (PLA) is a material traditionally used in fracture fixation because it does not last longer than 12–24 months. Some additives can lead to negative changes in degradation (*e.g.*, xanthan gum), whereas others, including citrate ions, hyaluronic acid, and collagen, can regulate the rate of degradation and stimulate osteoblast-like activity, cell adhesion, and bone formation.^111^

## 5. Applications polymeric nanocomposites in tissue engineering

The practical value of polymeric nanocomposites in tissue engineering is ultimately determined not by their laboratory properties alone, but by their ability to address the specific mechanical, biological, and clinical requirements of each target tissue. This section provides critical evaluation of the five most clinically relevant application areas such as bone, cartilage, skin, neural, and vascular tissue engineering. For each application, the following framework is applied: (i) the mechanical and biological demands of the target tissue; (ii) a critical comparative analysis of the polymer systems that have been studied; (iii) identification of the most clinically advanced approaches and the evidence behind them; and (iv) an explicit analysis of the translational bottleneck; the specific barrier that currently prevents the most promising scaffold systems from reaching patients. Overview of the application of polymeric nanocomposites in tissue engineering is discussed in Table 3.

**Table 3:** Use of polymeric nanocomposites in tissue engineering applications

S. No	Objective	Methodology	Polymer used	Outcome	Translational status	Primary limiting factor
1	β-TCP-PCL nanocomposites for bone implants	Cold sintering	PCL	Improved mechanical properties and biocompatibility^112^	Preclinical	Scale-up and sterilisation compatibility
2	Graphene/HAp for bone TE	Self-assembly	Graphene, HAp	Enhanced mechanical strength and bioactivity^113^	Preclinical	CNT/graphene long-term biocompatibility data absent
3	Bacterial cellulose–collagen for bone TE	Crosslinking	Bacterial cellulose, collagen	Improved mechanical properties^114^	Preclinical	Batch-to-batch variability in bacterial cellulose
4	nHAp/alginate/chitosan composite	Freeze-drying	HAp, alginate, chitosan	Enhanced structural properties for bone^115^	Preclinical	Insufficient compressive strength for cortical bone
5	Gelatin–silica nanofibrous scaffolds	TIPS	Gelatin, silica	ECM-mimicking architecture^116^	Preclinical	Weak mechanical strength under physiological load
6	CS-gelatin-alginate-HAp scaffold	Fabrication and characterisation	Chitosan, gelatin, alginate	Improved mechanical properties and biocompatibility^117^	Preclinical	Regulatory uncertainty for multi-component natural polymer systems
7	nHAp on PLGA/PLGA-collagen	Electrospinning	PLGA, collagen	Promoted osteoblastic behaviour^118^	Preclinical	Acidic PLGA degradation products at defect site
8	nHAp/polymer composite scaffolds	TIPS	nHAp/polymer	Improved biocompatibility and mechanics^119^	Preclinical	IVIVC model absent for nHAp composites
9	CNT-coated bioglass scaffolds	Electrophoretic deposition	CNTs	Improved electrical conductivity and bioactivity^120^	Preclinical	CNT cytotoxicity and long-term biopersistence
10	Injectable PPF nanocomposites	Crosslinking	Poly(propylene fumarate)	Cytocompatibility and mechanical integrity^121^	Preclinical	Injection viscosity limits clinical practicality
11	Chitosan scaffold for cartilage wound healing	Ionic and physical gelation	Chitosan	Chondrocyte attachment and matrix production^122^	Preclinical	Insufficient compressive modulus for load-bearing sites
12	ADMSCs on PDLCL for cartilage repair	Cell culture and implantation	Poly(dl-lactide-ε-caprolactone)	Mature chondrocytes observed in rats^123^	Preclinical	No large animal or human data
13	Bilayer collagen sponge for cartilage	Freeze-drying and layering	Type I collagen	Clinical improvement at 24 months^124^	Clinical use (CE-marked)	High cost; availability limited to specialised centres
14	Alginate nanofibers for ECM mimicry	Electrospinning	Alginate-PEO	Improved fibre formation and strength^125^	Preclinical	Low mechanical strength of alginate fibres
15	Silk fibroin hydrogels for cartilage	Sol–gel transition	Silk fibroin	Higher cartilage ECM deposition^126^	Preclinical	Batch variability in silk fibroin source
16	PLGA-GCH hybrid hydrogels	Composite preparation	PLGA-GCH	Increased MSC proliferation and GAG synthesis^127^	Preclinical	Complex formulation limits reproducible scale-up
17	Hyaluronic acid hydrogels for cartilage	Photo-crosslinking	Hyaluronic acid	Enhanced chondrocyte proliferation^128^	Clinical trials ongoing	Rapid enzymatic degradation *in vivo*
18	Injectable HA-MA hydrogels	Photo-crosslinking	HA-methacrylate	Enhanced cartilage matrix production *in vivo*^129^	Preclinical	Residual photoinitiator cytotoxicity
19	pColHAp/ChS composite scaffold	Freeze-drying	Collagen, HAp, chondroitin sulfate	Enhanced compressive strength^130^	Preclinical	Long-term mechanical stability unclear
20	Gelatin-GTA/GP scaffold for cartilage	Gel-crosslinking	Gelatin + crosslinker	Chondrogenesis and ECM secretion supported^131^	Preclinical	GTA cytotoxicity at higher concentrations
21	PVA/dextran-aldehyde hydrogel for wound healing	Freeze–thaw/lyophilisation	PVA/dextran-aldehyde	High tensile strength, minimal cytotoxicity^132^	Preclinical	Non-biodegradable PVA component
22	Lignin-chitosan-PVA scaffold for wound healing	Blending	Lignin-CS-PVA	High mechanical strength, re-epithelialisation^133^	Preclinical	Lignin batch variability; regulatory novelty
23	HA/poloxamer hydrogel for wound healing	Self-assembly	HA/Poloxamer	Improved angiogenesis and re-epithelialisation^134^	Preclinical	Poloxamer non-biodegradability
24	3D-printed chitosan scaffolds + growth factors	3D printing	Chitosan	Cartilage-specific ECM production^135^	Preclinical	3D printing resolution limits for soft hydrogels
25	GO-chitosan hydrogels for neural TE	Sonication/casting	GO and chitosan	Enhanced nerve cell growth by 20%^136^	Preclinical	GO long-term *in vivo* safety not established
26	PANI/PCL/gelatin nanofibers for neural TE	Electrospinning	PANI, PCL, gelatin	Enhanced neurite outgrowth upon electrical stimulation^137^	Preclinical	PANI non-biodegradability; regulatory uncertainty
27	CNF scaffolds for neural stem cells	Electrospinning + thermal treatment	PAN/CNF	Neural stem cell differentiation with electrical stimulation^138^	Preclinical	CNF cytotoxicity at high concentration
28	PPy/cellulose conductive hydrogels	*In situ* polymerisation	PPy with cellulose	Enhanced PC12 viability and neurite extension^139^	Preclinical	PPy non-biodegradability
29	PPy/alginate hydrogels for neural TE	Ionic crosslinking	PPy and alginate	Improved hMSC neural differentiation *in vivo*^140^	Preclinical	Two-year large animal biocompatibility data absent
30	Collagen conduits for peripheral nerve	Standard implantation	Collagen	Improved nerve regeneration in non-human primates^141^	FDA-cleared (NeuraGe, NeuroMatrix)	Gaps >3 cm; CNS applications not addressed
31	Silk fibroin scaffolds for neural repair	Electrospinning	Silk fibroin	Neurite outgrowth and axonal regeneration^142^	Preclinical	Batch variability; *in vivo* degradation rate inconsistent
32	Gelatin scaffolds for nerve repair	Electrospinning	Gelatin	Improved neural differentiation and axon guidance^143^	Preclinical	Poor mechanical stability of gelatin conduits *in vivo*
33	Alginate scaffolds for neural TE	Covalent crosslinking	Alginate	Axonal elongation and functional recovery^144^	Preclinical	Low mechanical strength; rapid degradation
34	Chitosan scaffolds for nerve regeneration	Electrospinning/freeze-drying	Chitosan	Schwann cell adhesion and neurite outgrowth^145^	Preclinical	Insufficient mechanical properties for long conduits
35	Small-diameter vascular grafts	Electrospinning	PCL, PLGA, collagen, elastin	Improved endothelialisation and mechanical stability^146^	Preclinical	Thrombosis risk; PMA regulatory requirement
36	3D-printed vascular scaffolds	3D bioprinting	GelMA, pluronic F127	Perfusable vascular structures^147^	Preclinical	Insufficient long-term mechanical integrity
37	Collagen-elastin vascular scaffolds	Collagen-elastin assembly	Collagen, elastin	Improved contractility and organised collagen fibers^148^	Preclinical	Rapid enzymatic degradation in high-flow arterial positions
38	Silk grafts for vascular TE	Electrospinning	Silk fibroin	Rapid endothelialisation and high patency rates^149^	Preclinical	Patency not consistently achieved in all models
39	Chitosan/PLCL artery scaffolds	Lyophilisation	Chitosan, PLCL	Artery-like regeneration in canine models^150^	Preclinical	Limited to low-pressure venous equivalents
40	Polyurethane vascular grafts	Electrospinning	Polyurethane	High compliance and endothelialisation to 6 months in rats^151^	Preclinical	Insufficient cellularisation of vascular wall long-term
41	PGA/PLGA/PCL composite grafts	Electrospinning/electrospraying	PGA, PLGA, PCL	Collagen-rich grafts with enhanced strength^152^	Preclinical	Rapid PGA degradation compromises early mechanical integrity
42	Triple-layer silk/PAM/TPU graft	Electrospinning/hydrogel assembly	Silk, PAM, TPU	Improved mechanical strength and suture retention^153^	Preclinical	Liquid leakage; insufficient elasticity at anastomosis
43	Poly(1,3-trimethylene carbonate) elastic porous graft	Phase inversion/self-assembly	Poly(TMC)	Suture retention and mechanical similarity to human arteries^154^	Preclinical	Long-term degradation behaviour under pulsatile flow not established
44	PLGA/collagen/elastin scaffolds	Dual electrospinning	PLGA, collagen, elastin	High endothelial cell attachment and mechanical compliance^155^	Preclinical	Collagen and elastin degradation rate mismatch with neotissue formation

### 5.1 Bone tissue engineering

Bone is a natural nanocomposite itself, consisting of approximately 70% nanocrystalline hydroxyapatite (nHAp) embedded within an organic collagen matrix. Nanocrystalline hydroxyapatite, which measures approximately 20–80 nm in length and 2–5 nm in width, provides stiffness and compressive strength while collagen fibrils contribute the flexibility and fracture toughness necessary for the skeleton to bear dynamic physiological loads; it is this inorganic–organic hierarchical combination that any synthetic scaffold must replicate to function successfully in bone defect repair. The mechanical requirements are stringent: compressive strength of 100–230 MPa for cortical bone and 2–12 MPa for cancellous bone, with elastic moduli of 15–25 GPa and 0.1–5 GPa respectively. Scaffolds must also be osteoinductive, osteoconductive, and sufficiently porous (pore size 100–500 µm, porosity >70%) to allow cell infiltration, vascularisation, and bone ingrowth.^156,157^

Among all nanocomposite systems studied for bone tissue engineering, PLGA/nano-hydroxyapatite composites have demonstrated the strongest convergence of preclinical evidence and regulatory feasibility. PLGA/nano-HA scaffolds cultivated with mesenchymal stem cells demonstrated larger cell counts, improved cell adherence, increased cell proliferation, and better alkaline phosphatase activity compared with PLGA control scaffolds; at 21 days after implantation into rabbit mandibular defects, these scaffolds showed early bone trabeculae formation, and at six weeks they tested positive for bone markers osteonectin and osteopontin indicating active bone deposition. The success of this system is not coincidental. HA's chemical resemblance to natural bone mineral gives it intrinsic osteoconductive activity that cells recognise without additional surface functionalisation; PLGA's co-polymer ratio allows degradation timing to be matched to the bone healing timeline; and both materials have established FDA regulatory precedent that substantially reduces the pre-market approval burden compared with novel materials.^158–160^ Composites reinforced with carbon nanotubes and graphene exhibit outstanding mechanical characteristics but lack equivalent clinical development. This contrast is informative; it is not a result of poor scientific merit but a lack of an established safety record. Nanocomposites functionalized with HAp containing graphene and CNTs exhibit improved drug loading and controlled release kinetics in animal models, where osteoblast viability exceeds 90% in many cases; yet the difficulties of scale-up, reproducibility, and clinical validation have thus far hindered their development beyond preclinical testing.^161^ Injectable nanocomposite systems represent an important recent development for minimally invasive bone repair. GelMA-HAp-nanosilicate ternary composite hydrogels, prepared by *in situ* crosslinking with embedded mesenchymal stem cells, showed faster bone formation at eight weeks in a rat calvarial defect model compared with GelMA alone, GelMA-HAp, and GelMA-SN controls demonstrating that the synergistic combination of a mineralisation-promoting filler (HAp) and a bioactive silicate phase produces superior outcomes to either component in isolation.^162^

The primary barrier to clinical translation in bone tissue engineering is the absence of standardised *in vitro-in vivo* correlation (IVIVC) models that are accepted by regulatory agencies. Polymeric composite scaffolds for bone tissue engineering perform differently in small-diameter defect models compared with large-diameter defects, and these differences in mechanical loading environment, vascularisation capacity, and host immune response mean that results from rodent critical-size defect models routinely fail to predict outcomes in larger animal or human applications. Until IVIVC models for bone scaffolds are validated, the preclinical evidence base will remain structurally limited in its predictive value, adding cost and time to every clinical development programme.^163^

### 5.2 Cartilage tissue engineering

Articular cartilage is one of the most clinically and scientifically challenging tissue engineering targets because it is simultaneously avascular, aneural, largely acellular, and subjected to continuous mechanical loading. Its intrinsic regenerative capacity is minimal because the absence of blood supply means that progenitor cells cannot migrate to the injury site in meaningful numbers. Approximately 15 million cartilage injuries occur annually in the USA alone, and conventional surgical interventions; microfracture, autologous chondrocyte implantation, and osteochondral allografts frequently fail to restore native articular function, particularly in large defects.^164^ However, the major overlooked fact about cartilage tissue engineering is that the sole use of hydrogel scaffolds fails to satisfy mechanical requirements for weight-bearing tissues. In cases of weight-bearing tissues such as cartilages in the knee joint, hydrogels have to be tough, strong, and capable of dissipating sufficient energy. Single network hydrogels usually employ a high cross-linking density mechanism but cannot achieve the multiple mechanical requirements at once, prompting the creation of advanced hybrid structures like double network hydrogels and micro-nano composite hydrogels, wherein the nanoparticles act as multifunctional cross-linking agents to enhance modulus and toughness. Compressive moduli of single-component hydrogels typically range from 10–100 kPa, orders of magnitude below the GPa-range stiffness of native articular cartilage under impact loading; a mechanical mismatch that explains the persistent failure of hydrogel-only approaches *in vivo* despite their consistently encouraging *in vitro* results.^165,166^

Nanocomposite hydrogels have begun to address this gap. Incorporating graphene oxide nanosheets into sodium alginate ion networks has produced composite hydrogels with a modulus of 198 GPa and tensile strength of 1215 MPa, while carbon dots incorporated as crosslinkers into polymer hydrogels enhance the storage modulus, loss modulus, viscosity, and lubricating properties directly relevant to reducing friction at the articular surface in load-bearing applications. For injectable applications specifically, thermosensitive polymer nanoparticle-based hydrogels that form *in situ* upon injection at body temperature, and that carry dual-interacting surfaces for growth factor capture and sustained release, have demonstrated successful *in vivo* bone morphogenetic protein-2 release and new bone layer formation at cranial sites; a strategy that is directly translatable to injectable cartilage repair applications.^167,168^ Zonally structured, gradient scaffolds represent the most scientifically sophisticated direction in cartilage tissue engineering.^169^ Recent research has focused on developing multilayered hydrogel scaffolds to mimic the zonal organisation of native cartilage tissue, with gradients in mechanical properties, ECM composition, and bioactive factors across layers to guide cell differentiation and tissue formation; trilayered hydrogel constructs with varied degradation rates across layers have been shown to facilitate sustained cell infiltration and ECM formation by replicating the native tissue gradient from cartilage to subchondral bone.^170^

The critical challenge for cartilage tissue engineering is achieving zonally structured scaffolds that replicate the depth-dependent composition of native articular cartilage including the distinct collagen fibre orientation, proteoglycan content, and chondrocyte phenotype of the superficial, middle, and deep zones at clinically relevant scales using reproducible, GMP-compatible fabrication methods. Despite significant progress in nanocomposite hydrogel design, the developed systems are still far from native cartilage in terms of physicochemical properties and structural geometry, and there is a notable scarcity of studies reporting the *in vivo* performance of newly developed hydrogel formulations, indicating that further *in vivo* validation is urgently needed before clinical translation can be considered.^170,171^

### 5.3 Skin tissue engineering

Skin is the body's largest organ and its primary barrier against the external environment. Severe skin loss from burns, chronic ulcers, or traumatic wounds represents a life-threatening clinical emergency for which autologous skin grafting remains the gold standard but is severely limited by donor site availability. Tissue-engineered skin substitutes must replicate the bilayer architecture of native skin, support cell adhesion, migration, and differentiation, resist wound contraction and infection, and eventually integrate with the host vasculature. Skin tissue engineering has produced the most clinically translated results of all tissue types, driven by the accessibility of the implant site and the clinical urgency of severe burn treatment. Among polymeric nanocomposites, chitosan-based systems offer a unique and practical advantage that synthetic alternatives cannot replicate: intrinsic antimicrobial activity. Polyester-based synthetic materials such as PCL and PLGA, which have received FDA approval for skin tissue engineering applications, have been successfully used in nanocomposite hydrogels that exhibit injectable, self-healing behaviour, robust antibacterial activity, and the ability to promote angiogenesis and neovascularisation demonstrating that the anti-infection and pro-vascularisation properties of a scaffold system can be combined in a single nanocomposite platform.^172–174^

Spatiotemporally controlled growth factor delivery within nanofiber scaffolds represents the most sophisticated recent development in skin tissue engineering. A PCL-based nanofiber scaffold loaded with PDGF-BB, VEGF, and EGF in a spatial and temporal pattern aligned with the overlapping phases of wound healing haemostasis/inflammation, proliferation, and remodelling demonstrated enhanced wound closure and modulation of the wound microenvironment through activation of PI3K-Akt, MAPK, and immune pathways in a preclinical porcine model, confirming that stage-specific growth factor delivery from nanocomposite scaffolds can outperform passive release systems.^175^ The critical comparison between natural and synthetic polymer systems in skin tissue engineering reveals a clear divergence in their clinical niches: natural materials such as collagen and gelatin contain RGD motifs that strongly promote cell adhesion and proliferation but their rapid degradation and poor mechanical stability restrict their use to luminal coatings or composite layers rather than standalone load-bearing scaffolds; chitosan, owing to its cationic nature, enhances cell adhesion and exhibits antibacterial properties but also requires crosslinking or blending with other materials to achieve adequate mechanical strength; while synthetic materials possess excellent mechanical strength but their bioinert surfaces result in insufficient endothelialization and require surface functionalisation to reduce the risk of adverse reactions.^176,177^

Vascularisation remains the dominant barrier to full-thickness skin regeneration with polymeric nanocomposite scaffolds.^178,179^ Engineered skin constructs thicker than approximately 200 µm develop hypoxic cores that limit cell viability and impair integration with host vasculature. Zinc oxide nanoparticles incorporated into PCL electrospun membranes have shown increased dermal fibroblast cell proliferation and upregulated expression of the angiogenic factors FGF2 and VEGF-A, demonstrating that nanoparticle incorporation into scaffold matrices can actively promote the vascularisation response but this approach has not yet achieved consistent clinical results in full-thickness wounds. Cerium oxide nanoparticle-based scaffolds and VEGF-loaded composite hydrogels are among the most promising pre-vascularisation strategies, but their clinical translation requires resolution of dose–response optimisation and standardisation of growth factor incorporation methods that are currently variable across studies.^180,181^

### 5.4 Neural tissue engineering

Neural tissue engineering targets one of medicine's most intractable regenerative challenges. The peripheral nervous system (PNS) has limited intrinsic regenerative capacity and completely fails over nerve gaps greater than 3 cm; the central nervous system (CNS) has virtually no regenerative capacity due to the inhibitory microenvironment created by glial scarring. Scaffolds for neural tissue engineering must provide not only structural support but also electrical conductivity, appropriate mechanical compliance matching the Young's modulus of neural tissue (0.1–1 MPa), topographical guidance cues for axonal growth, and controlled delivery of neurotrophic factors.^182,183^ The field of neural tissue engineering presents an instructive clinical paradox: the materials that perform best in laboratory models are not the ones that have succeeded clinically. The FDA has only approved nerve conduits for peripheral nerve repair, while translational products addressing more complex neurological issues remain minimal; this regulatory position reflects the conservative safety paradigm applied to CNS-adjacent devices, where long-term biocompatibility data for novel conductive nanomaterials over multi-year implantation periods does not yet exist.^184^ Conductive nanocomposite hydrogels incorporating CNTs, graphene derivatives, conductive polymers such as PEDOT and polypyrrole, or metallic nanoparticles represent the most actively researched class of neural scaffold materials. A systematic scoping review of 125 studies on conductive nanocomposite hydrogels for neural tissue engineering found that carbon-based nanomaterials (CNTs, graphene derivatives) dominated at 36.8% of systems, followed by metals (iron oxides, gold) at 24.0% and conductive polymers at 16.0%; for CNS repair applications, antioxidant-conductive hybrids and immunomodulatory systems showed the greatest efficacy by mitigating oxidative stress and neuroinflammation, while for peripheral nerve repair, biomimetic scaffold design and stimuli-responsive strategies demonstrated the most promising functional outcomes.^185^ In peripheral nerve repair specifically, 3D PCL/CNT composite scaffolds have shown measurable functional recovery. Three-dimensional conductive PCL/CNT nerve scaffolds fabricated by integration moulding, when combined with electrical stimulation that mimicked endogenous electric fields, significantly promoted Schwann cell proliferation and neurotrophic factor gene expression *in vitro*, and in a 15 mm rat sciatic nerve defect model *in vivo*, functional and histological tests confirmed that these scaffolds effectively promoted nerve regeneration and functional recovery. However, collagen conduits specifically NeuraGen and NeuroMatrix remain the only commercially available and FDA-cleared polymeric nerve repair devices, and their success reflects established safety data and compatibility with existing surgical workflows rather than superior *in vitro* performance compared with conductive nanocomposites.^186,187^ Long-term biocompatibility of conductive nanofillers in neural applications is incompletely characterised. Carbon nanotubes and graphene have been reported to cause inflammation, fibrosis, oxidative stress, and DNA damage in murine models, and the safety of conductive polymers such as PEDOT and polypyrrole has not yet been fully established for long-term neural implants; regulatory agencies require two-year implantation data in relevant animal models before IDE applications for Class III neural devices, and no conductive polymeric nanocomposite system has yet completed this qualification.^188,189^

### 5.5 Vascular tissue engineering

Cardiovascular disease remains the leading cause of global mortality, creating a critical and unmet clinical demand for small-diameter (<6 mm) arterial grafts where existing synthetic prosthetics expanded polytetrafluoroethylene (ePTFE) and polyethylene terephthalate (PET/Dacron) fail at clinically unacceptable rates due to thrombosis and intimal hyperplasia. Large-calibre synthetic grafts are well-established in clinical practice, but these synthetic materials fail in small-diameter applications due to thrombosis and intimal hyperplasia, and autologous grafts are constrained by limited availability and variable quality. Vascular scaffolds must replicate the trilayer architecture of native arteries such as intima, media, and adventitia withstand pulsatile haemodynamic loading, promote rapid endothelialisation to prevent thrombosis, and degrade at a rate matched to neotissue formation.^190^ Among all synthetic polymer systems evaluated for small-diameter vascular grafts, electrospun PCL-based scaffolds have demonstrated the strongest combination of mechanical performance and preclinical evidence. A PCL-based scaffold small-diameter vascular graft with 2 mm inner diameter produced by electrospinning showed a burst pressure of approximately 3280 mmHg, comparable to native internal mammary artery values and exceeding those of saphenous vein demonstrating that electrospun PCL can produce mechanically competent vascular substitutes. PCL's slow degradation (2–4 years) provides long-term structural support during the neotissue formation process, and its processability by electrospinning enables the creation of fibre architectures that mimic the orientation of native collagen in arterial walls.^191^ Hybrid natural-synthetic polymer systems have shown particular promise in translational animal models. Cell-free electrospun PCL/chitosan nanofiber vascular grafts with a 5 mm diameter demonstrated 100% patency in mice as infrarenal aorta interposition grafts at six months, and when tested as carotid artery grafts in sheep a large animal model more predictive of human outcomes the grafts showed acceptable patency, supporting the use of fast-degrading hybrid nanofiber scaffolds that promote host cell infiltration and neotissue formation rather than serving as permanent mechanical substitutes.^192^ The clinical translation landscape for vascular TEVGs has advanced measurably in recent years. The most recent tissue-engineered vascular graft to enter clinical trial phase is a POSS-PCU small-diameter graft currently being tested as arteriovenous fistulas for haemodialysis, with a study that began in 2021 and is expected to complete in 2025; this graft has a small diameter of less than 5 mm and is specifically evaluating patency rates at 18 months in patients with increased thrombosis risk representing a meaningful step toward clinical validation for synthetic small-diameter vascular grafts.^193^ The dominant barrier for vascular nanocomposite grafts is not technical performance but regulatory pathway length. Small-diameter vascular grafts are classified as Class III devices by the FDA, requiring Pre-Market Approval (PMA); a pathway that imposes costs exceeding $50 million and timelines exceeding ten years from design to market, compared with less than $500 000 and two to three years for 510(k)-cleared devices. Biodegradable polymers provide tunable degradation properties and better compliance, with PCL offering prolonged structural support due to its slow degradation, while PLGA degrades more rapidly to facilitate tissue remodelling but the acidic degradation by-products of PLGA-based systems may induce local inflammation that compromises endothelialisation and long-term patency. Until a predicate device pathway becomes available or the FDA establishes a dedicated *De Novo* pathway for polymeric nanocomposite vascular grafts, clinical translation will remain severely rate-limited regardless of preclinical performance.^194–196^

## 6. Challenges and future prospects

A critical assessment of the field requires not only an account of what polymeric nanocomposite scaffolds can do under ideal conditions, but an honest analysis of the obstacles that prevent laboratory performance from reaching patients. The following subsections address the most significant scientific, technological, and regulatory challenges facing the field, while also identifying the directions with the greatest potential to resolve them.

### 6.1 Stability and degradation control

Clinical use of any polymeric scaffold is highly dependent on the ability to degrade at the same rate as tissue healing; a seemingly straightforward design criterion that is challenging to accomplish in reality. Polymeric materials like polyethylene and silicone resist degradation within the biological milieu over years or even decades, which makes them ideal for permanent implants but not tissue engineering where scaffold degradation and subsequent tissue regeneration are needed.^197^ Biodegradable polymers including polylactide (PLA), polyglycolide (PGA), and their copolymer PLGA are degraded through three principal mechanisms: hydrolytic chain scission, oxidative attack by reactive oxygen species (ROS), and enzymatic cleavage. Natural polymers degrade through combinations of hydrolysis, erosion, solubilisation, and enzymatic breakdown, all of which are influenced by environmental conditions and the polymer's own properties including its ionic interactions; for example, gelatin is particularly susceptible to enzymatic degradation by collagenase and lysozyme; a characteristic that can be an advantage in some wound healing applications but a liability in structural bone scaffolds where enzymatic activity is unpredictable.^7,198^

The rate of degradation is governed by multiple interacting variables: polymer crystallinity, molecular weight, hydrophilicity, fabrication architecture (surface-to-volume ratio), and local physiological conditions including pH, temperature, and mechanical loading. Electrospun polymer membranes degrade faster than cast films of the same polymer because their much higher surface-area-to-volume ratio accelerates water absorption and hydrolysis; this means that degradation characterisation performed on bulk or cast samples substantially underestimates the *in vivo* degradation rate of electrospun scaffold constructs; a systematic error that has led to premature mechanical failure of otherwise well-designed scaffold systems in preclinical studies.^199,200^

A particularly important and underappreciated translational problem is that most published degradation studies are conducted under static *in vitro* conditions without mechanical loading. Flow perfusion substantially accelerates hydrolytic degradation of PCL-PLGA based scaffolds compared to static incubation, because fluid flow renews the local acidic degradation product concentration rather than allowing it to accumulate; this means that static degradation data systematically underestimates *in vivo* degradation rates, and degradation studies conducted in perfusion bioreactors are therefore more predictive of clinical performance than static immersion tests. Until standardised dynamic degradation testing protocols are adopted by the field and accepted by regulatory agencies, degradation data from static studies will continue to produce optimistic predictions that do not hold up *in vivo*.^201^

Critically, matching degradation rate to tissue healing rate requires not just selecting a polymer with an appropriate bulk degradation timeline, but engineering the composite so that degradation rate is maintained within the required window throughout the healing process. Scaffolds with intermediate degradation rates retaining approximately 60% of their molecular weight at 28 days; best enable gradual replacement of the polymer network with cell-derived ECM, because fast-degrading scaffolds lose structural integrity before sufficient ECM has formed to support the regenerating tissue, while slow-degrading scaffolds persist too long and block complete remodelling; this intermediate degradation behaviour represents the practical design target for most tissue engineering applications.^202^

### 6.2 Immune response

Scaffold implantation initiates an immediate, stereotyped cascade of innate immune responses that profoundly determines whether the scaffold integrates successfully or is rejected through fibrotic encapsulation. After implantation, biomaterials trigger a foreign body response characterised by the involvement of several cellular populations; macrophages are central to this response as highly plastic cells specialised in phagocytosis and capable of secreting cytokines that stimulate the proliferation and differentiation of the monocytic population and they can polarise into either a pro-inflammatory M1 phenotype, which releases pro-inflammatory cytokines and clears bacteria and debris, or an anti-inflammatory M2 phenotype, which promotes tissue healing and angiogenesis through the secretion of anti-inflammatory cytokines and growth factors.^203,204^

The clinical outcome of scaffold implantation is largely determined by the trajectory of macrophage polarisation over time. Advances in tissue engineering demonstrate that macrophage polarisation; the transition from pro-inflammatory M1 to anti-inflammatory M2 phenotypes can be influenced by scaffold material properties including surface chemistry, architecture, and stiffness to mitigate the foreign body response and enhance tissue regeneration; scaffold surface chemistry and structural features are therefore not merely biological compatibility considerations but active immunomodulatory design parameters that must be engineered deliberately for each tissue application.^205^

The regulatory consequences of persistent M1 macrophage activity are well-defined and clinically serious: chronic M1 dominance produces persistent inflammation, progressive fibrotic encapsulation of the scaffold, impaired vascularisation, and ultimately scaffold rejection. The balance between M1 and M2 macrophage activity determines whether the device undergoes successful integration with tissue repair and regeneration, or rejection with massive fibrous encapsulation and a constant failure rate of approximately 10% for all implantable devices due to foreign body response represents a cost of US $10 billion per year globally, underlining the scale of the clinical problem that remains unsolved by current scaffold designs.^206^

**Fig. 4:**
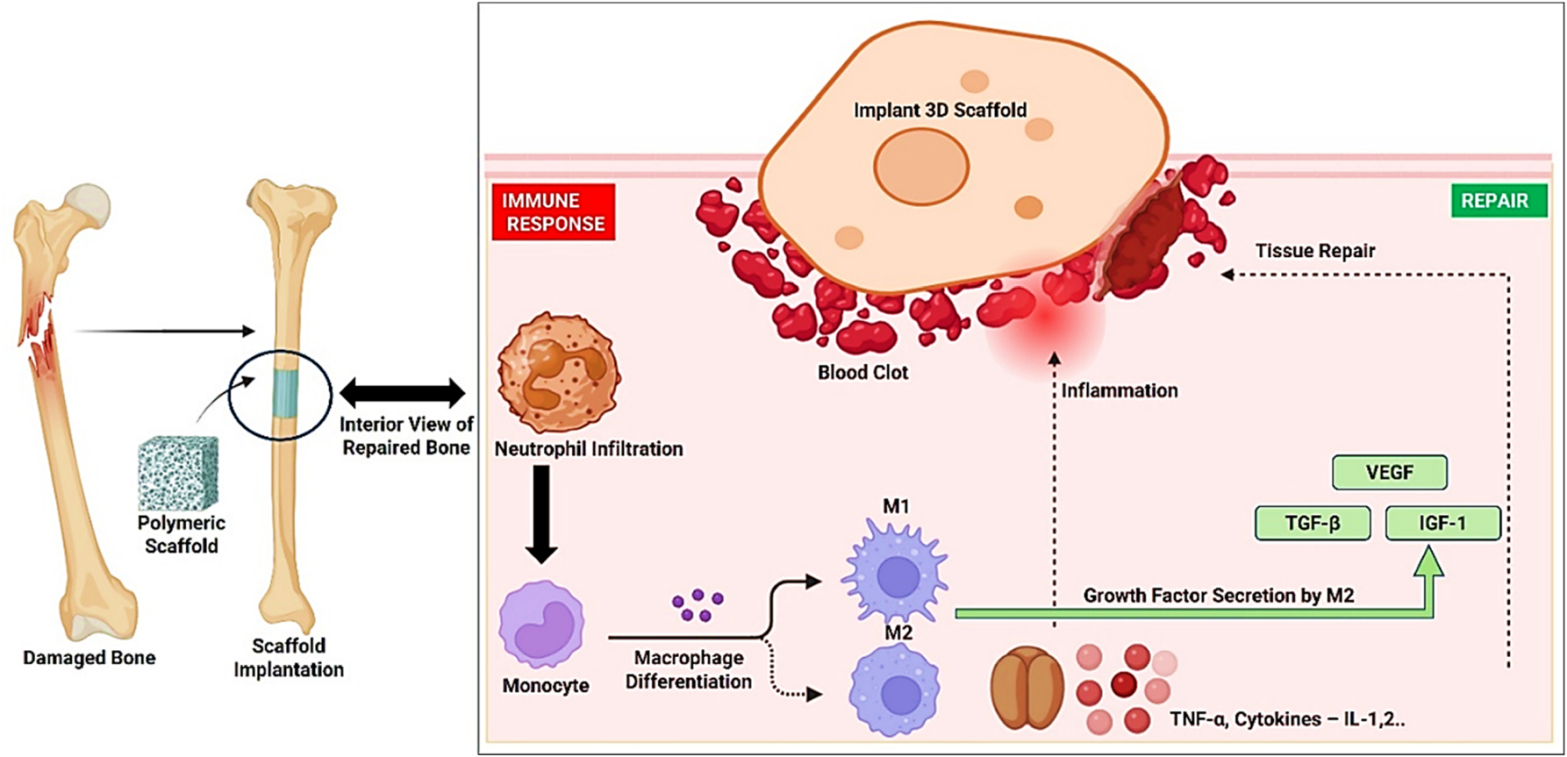
Illustration of the immune response to polymeric scaffold implantation for bone repair, highlighting the sequence of inflammatory events, including neutrophil infiltration, macrophage differentiation into M1 and M2 phenotypes, secretion of pro- and anti-inflammatory cytokines (TNF-α, IL-1, IL-2), and the role of growth factors (VEGF, TGF-β, IGF-1) in promoting tissue repair and integration. The images were created with Biorender.com.

Recent strategies for engineering immune-tolerant scaffolds include the controlled release of anti-inflammatory cytokines (IL-4, IL-10) and growth factors from scaffold matrices, surface modification with immune-modulating molecules (zwitterionic polymers, polyethylene glycol), and architectural optimisation of pore size to guide macrophage phenotype. Regulatory T cell-conditioned medium loaded onto decellularised ECM scaffolds effectively promoted M2 macrophage polarisation and inhibited M1 polarisation under inflammatory conditions both *in vitro* and *in vivo*, demonstrating that the immunological microenvironment of the scaffold can be shifted from rejection-promoting to regeneration-promoting through bioactive loading; a strategy that is directly translatable to nanocomposite scaffold platforms where controlled release mechanisms are already available. Additionally, 3D scaffolds microarchitecture itself; independent of chemical composition profoundly influences macrophage cytoskeletal organisation and metabolic activity, with pore geometry directly modulating the balance between M1 and M2 polarisation; this means that architectural design decisions are simultaneously mechanical and immunological design decisions, and must be considered together rather than sequentially.^160,207^ Schematic representation of the immune response following polymeric scaffold implantation for bone repair is given in Fig. 4.

### 6.3 Scale-up and reproducibility

The shift from synthesis under laboratory conditions to manufacturing under GMP standards is perhaps the most consistently underestimated problem in the clinical translation of polymeric nanocomposites. Synthesis under laboratory conditions is usually associated with superb nanoparticle dispersion, but this process relies upon mixing parameters which cannot be simply scaled-up in the context of industrial manufacturing volumes. Batch-to-batch variability in nanoparticle size distribution, dispersion homogeneity, and polymer molecular weight distribution can produce scaffolds with substantially different mechanical properties and biological performance despite nominal compositional identity; a problem that is difficult to detect with the limited characterisation typically performed in academic research settings but that is unacceptable in a GMP manufacturing context.^208^

Microfluidics-based approaches have emerged as a low-cost, reproducible, and scalable platform for the manufacturing of biomimetic scaffolds for tissue engineering, offering precise control over nanoparticle properties, uniform mixing in laminar flow regimes, and process parameters that are well-characterised and readily transferred between laboratories.^209^ Specifically, microfluidic synthesis of PLGA nanoparticles has been demonstrated at production rates of 1300 mg per hour with size reproducibly controlled between 50 and 150 nm; a throughput compatible with clinical trial material production by parallelising microchannels within a single pressure-tolerant device. These scale-up strategies show concrete potential to meet clinical or industrial production volumes *via* microfluidic synthesis, overcoming the fundamental limitation of batch processes where the scale of mixing equipment and precise details of reagent addition dramatically affect particle dispersity and size in ways that cannot be controlled at large scale.^166^

For the polymer matrix phase of nanocomposites specifically, melt mixing using twin-screw extruders is the only approach that is already compatible with industrial polymer processing infrastructure and capable of achieving the throughputs required for commercial manufacturing. Solution mixing and *in situ* polymerisation achieve superior nanoparticle dispersion at small scale but cannot be practically scaled to industrial volumes. The choice of scale-up method therefore involves a direct trade-off between dispersion quality and production feasibility; a trade-off that must be explicitly addressed in the design of any scaffold system intended for clinical translation.^210^

### 6.4 Stimulus-responsive and 4D nanocomposites

The development of stimulus-responsive polymeric nanocomposites; materials that actively change their properties in response to physiological or externally applied cues represents one of the most significant recent advances in the field. These systems move scaffold design from the passive provision of a structural template to the active, dynamic modulation of the regenerative microenvironment. 4D bioprinting adds the functional dimension of time to 3D bioprinting by enabling printed scaffolds and structures to undergo predetermined, reversible deformations in response to specific environmental stimuli including light, magnetic fields, pH, humidity, and temperature; by incorporating intelligent biomaterials into the printing process, the construct gains the ability to change its shape, mechanical properties, or function after fabrication; a capability that is directly relevant to minimally invasive implantation and dynamic scaffold adaptation to tissue healing phases.^211^

#### 6.4.1 pH-responsive nanocomposites

The infected wound microenvironment is characterised by local acidification (pH 5.5–6.5 compared to healthy tissue pH 7.2–7.4), providing a natural, disease-specific chemical trigger for on-demand antimicrobial and anti-inflammatory agent release. Hydrogels composed of chitosan/ZnO composite materials have shown that ZnO nanoparticles can be dissolved and Zn^2+^ ions can be released at an acidic pH, thus effectively eliminating *S. aureus* and *P. aeruginosa* biofilms. However, the key problem of such systems lies in the heterogeneity of the pH value in chronic wounds. Therefore, the pH trigger should be carefully calibrated to ensure that the system does not become activated in the case of transient acidity in a healthy environment.^212^

#### 6.4.2 Thermoresponsive systems

Nanocomposites consisting of PNIPAAm exhibit a sudden, reversible change in their volume phase transition at their lower critical solution temperature (LCST, 32 °C). This property can be used for liquid injection followed by solidification *in vivo*, providing a means of scaffoldless implantation without the need for any predefined geometry for the scaffold itself. However, PNIPAAm's non-biodegradability remains a fundamental barrier to clinical adoption as a standalone material, and the narrow LCST window between ambient and body temperature means that minor temperature variations during storage, transport, or handling can trigger premature gelation. Recent work incorporating PNIPAAm as a minor thermoresponsive component within biodegradable PCL or PLA matrices aims to retain injectable functionality while enabling eventual scaffold clearance through polymer backbone degradation.^213^

#### 6.4.3 Magnetically responsive nanocomposites

Fe_3_O_4_ nanoparticle incorporation into polymer matrices enables remote mechanical actuation through externally applied magnetic fields, magnetically guided drug delivery, and hyperthermic bacterial clearance. In bone tissue engineering, magnetically responsive scaffolds exposed to external magnetic field stimulation can provide mechanical cues that mimic physiological loading and promote osteogenic and chondrogenic differentiation of stem cells without surgical intervention; a potentially transformative approach for non-invasive postoperative rehabilitation that directly addresses the limited loading environment of conventionally immobilised orthopaedic patients. The primary safety concern is MRI compatibility which has been substantially addressed through the use of ultra-small superparamagnetic iron oxide (USPIO) nanoparticles, which are non-perturbative at clinical MRI field strengths, though regulatory acceptance for individual composite systems remains on a case-by-case basis.^214^

#### 6.4.4 Photoresponsive systems

Azobenzene, spiropyran, and coumarin-functionalised scaffolds enable light-triggered conformational changes that modulate cell alignment, matrix stiffness, and contact guidance cues in spatially defined patterns. Light-responsive 4D bioprinted materials function by converting optical signals into physical and chemical responses when exposed to optical stimuli at high spatial resolution, polymer chains within the scaffold undergo photodegradation or photocrosslinking, leading to controlled shape deformations that can be used to guide axonal growth direction in neural tissue applications, trigger drug release from photocleavable linkers, or stiffen the scaffold matrix in specific zones to direct stem cell differentiation. The primary translational limitation is tissue penetration depth: UV and visible light penetrate only 1–5 mm in biological tissue, restricting photoresponsive scaffold use to superficial wounds, corneal repair, or endoscopic-access sites unless near-infrared (NIR)-responsive chromophores which penetrate 2–5 cm are employed.^215^

#### 6.4.5 4D bioprinting: convergence of smart materials and precision fabrication

4D bioprinting is defined as the 3D printing of stimulus-responsive materials that change shape or function after fabrication in response to a planned environmental trigger. 4D-printed shape memory polymers can promote spatiotemporally controlled architectures enabling minimally invasive implants, dynamic tissue scaffolds, and multifunctional drug delivery; current roadblocks to clinical implementation include cytotoxicity of photopolymerisable resin components, sterilisation compatibility of smart material systems, regulatory compliance uncertainty for time-variant devices, and device shelf-life management; challenges that are distinct from those of conventional scaffolds and require new regulatory frameworks. While 4D bioprinting is currently at Technology Readiness Level 3–4 (laboratory validation), it represents the horizon most likely to produce clinically transformative tissue engineering solutions within the next decade.^216,217^

### 6.5 Clinical translation and regulatory considerations

Even with many years of preclinical studies, there is still a huge difference between what works in laboratory settings and the clinical implementation of polymeric nanocomposite scaffolds. Those scaffold systems that have performed well in clinical settings are not the most advanced in technology but rather those that have the best safety records, regulatory history, and surgical integration. This section offers an honest discussion of the state of clinical reality and the regulations guiding it.^218^

Several polymeric scaffold systems have achieved regulatory approval or are in active clinical evaluation. Collagen-based nerve conduits (NeuraGen, NeuroMatrix) are FDA-cleared Class II devices under 21 CFR 888.3045 for peripheral nerve repair in gaps up to 3 cm.^219^ Acellular collagen/HA composites for articular cartilage repair have received CE marking in the EU (*e.g.*, Chondro-Gide, Geistlich Pharma). PLGA-based resorbable bone screws and interference screws are widely used in orthopaedic and sports medicine surgery. Electrospun PCL/collagen vascular grafts are in Phase I/II clinical evaluation in Europe (NCT04000438).^220^ The common factor across all of these is incremental improvement on a well-understood material platform, not radical novelty; a pattern that should inform the translational strategy of researchers developing nanocomposite systems.^221^

The FDA's 2023 guidance on nanotechnology-based medical devices requires specific characterisation data for nanocomposite devices: nanoparticle size distribution and agglomeration state under physiological conditions; leachable nanoparticle thresholds from the scaffold matrix; endotoxin testing per ISO 11135; and genotoxicity assessment for novel nanofillers per ISO 10993-3. The EMA's Committee for Advanced Therapies (CAT) evaluates nanocomposite scaffolds combined with cells as Advanced Therapy Medicinal Products (ATMPs), imposing substantially higher evidence thresholds including Phase III clinical trial data before marketing authorisation.^222^ Three regulatory-scientific barriers currently dominate the translation landscape: first, the absence of standardised long-term *in vivo* degradation models acceptable to both FDA and EMA creates a reproducibility gap between regulatory submissions; second, there is no consensus threshold for acceptable systemic nanoparticle exposure following scaffold implantation the field operates without any equivalent of the tolerable daily intake framework used for drug excipients; third, the absence of validated, standardised clinical outcome measures for tissue-specific endpoints (patient-reported outcome measures for cartilage repair, electrophysiological recovery metrics for nerve repair) limits the statistical power of small clinical trials and makes cross-trial comparison unreliable.^222^

### 6.6 Personalisation in tissue engineering

Patient-specific tissue engineering in which scaffold geometry, material composition, and biological loading are tailored to the anatomy, immunological profile, and clinical history of the individual patient represents the most clinically ambitious direction in the field. CAD/CAM processes are central to the clinical application of personalised 3D bioprinting, typically beginning with patient scanning using medical imaging modalities to generate 3D volumetric data in DICOM format, which is then processed through reverse engineering and surface model reconstruction to create a patient-specific scaffold geometry that precisely matches the defect to be repaired; an approach that improves anatomical fit and potentially reduces surgical intervention time compared with standard-sized implants.^223–225^

Personalisation addresses one of the most fundamental limitations of conventional tissue engineering: the mismatch between standard scaffold geometries and the irregular, patient-specific anatomy of actual clinical defects.^226^ Computationally optimised perfusion bioreactor-scaffold assemblies designed to match patient-specific calcaneal bone geometry using macroscale CFD-guided scaffold architectures achieved volumetric tissue growth of 13 mL approximately human calcaneus scale with pre-osteoblast cells at 7, 20, and 24 days in culture, demonstrating that the integration of computational modelling, patient-specific imaging, and 3D printing can enable the generation of clinically relevant tissue volumes that are not achievable with conventional scaffold designs.^227^ The personalised tissue-engineered vein (P-TEV) approach fabricating autologous vascular grafts from patient-derived cells seeded onto bioresorbable scaffolds matched to individual vascular anatomy exemplifies the clinical potential and the current limitations of personalised tissue engineering simultaneously: the individualised biological compatibility is excellent, but the manufacturing complexity, cost, and extended production time before implantation are substantial barriers to widespread adoption that have not yet been resolved by any currently available platform.^228,229^

### 6.7 Integration with bioprinting technologies

Polymeric bioinks have expanded the architectural complexity of tissue engineering scaffolds far beyond what conventional fabrication can achieve. Advanced biofabrication approaches include novel biomaterials which behave as living matter, being dynamically responsive and programmable for precise interaction with biological surroundings through the use of nanoscale scaffolds, responsive polymers, resorbable devices, and intelligent drug delivery systems in combination with additive manufacturing technologies to create programmable architectures with properties programmed down to the level of a voxel.^230,231^ The examples of recent developments in biomaterial engineering include self-assembled hydrogels composed of nucleotide lipids, able to organise into printable bioinks, pocket-sized 4D printers allowing for direct on-site wound bioprinting, and melt electro-writing techniques for production of reinforced hydrogel composite meshes. Of particular importance for scaffold printing is utilisation of sacrificial bioinks such as Pluronic F127, which allow for creation of perfusable vascular channels in printed constructs and thus help to overcome the problem of tissue vascularisation associated with the upper limit on construct thickness. Although considerable advances have been made in the field, there is still the challenge to produce human-size tissue constructs with preserved cell viability and mechanical stability, being the most imminent problem in practical implementation of bioprinting technologies.^232^

### 6.8 Integrating AI and machine learning in scaffold design

Artificial intelligence and machine learning have emerged as transformative tools for scaffold design optimisation, enabling the systematic exploration of high-dimensional materials parameter spaces covering composition, fabrication conditions, and biological outcomes simultaneously that are intractable by conventional one-variable-at-a-time experimental approaches. Bayesian optimisation (BO) is among the most effective ML strategies for tissue engineering applications, functioning as a sequential model-based optimisation technique that uses surrogate models to guide the search for optimal solutions; BO has been applied to multi-objective optimisation of variables for neotissue growth on 3D scaffolds in bioreactor settings, outperforming both random search and genetic algorithm approaches, demonstrating that model-guided optimisation substantially reduces the number of experiments needed to reach performance targets.^233–236^

#### 6.8.1 Supervised learning for property prediction

Artificial neural networks (ANNs), gradient boosting models, and random forest regressors have been applied to predict scaffold mechanical properties, degradation rates, and cell viability from materials composition and fabrication parameters. Entekhabi *et al.* employed ANNs and Kernel Ridge Regression (KRR) to forecast degradation rates of freeze-dried gelatin scaffolds crosslinked with genipin, achieving a mean squared error of 2.68%. However, ML models in tissue engineering often lack interpretability, which poses fundamental challenges for understanding the biological mechanisms underlying scaffold performance; ML techniques hold promise for optimising scaffold geometries but may not fully capture the complex, dynamic biological responses essential for effective tissue regeneration and inadequate or biased training data can result in models that fail to generalise to new scaffold designs.^237,238^

#### 6.8.2 Explainable AI for mechanistic insight

The interpretability limitation of black-box ML models is being addressed by Explainable AI (XAI) methods. XAI techniques such as SHAP (SHapley Additive exPlanations) values allow researchers to identify which scaffold material features are most predictive of biocompatibility and performance outcomes, providing not just predictions but mechanistic guidance that can inform rational materials design making the ML model a tool for scientific discovery rather than purely an engineering optimisation tool.^239–242^

#### 6.8.3 AI-optimised scaffold architecture

Beyond property prediction from existing designs, AI is now being used to generate novel scaffold architectures. Machine learning algorithms that predict and tailor lattice structure density and pore size to meet specific clinical requirements; optimising simultaneously for cell proliferation, nutrient exchange, mechanical integrity, and thermal stability under physiological conditions demonstrate that AI-guided architectural design can produce scaffolds that outperform conventional designs across multiple performance criteria simultaneously, a multi-objective optimisation that conventional design approaches cannot achieve efficiently.^239,243–245^

Some challenges currently limit broader ML adoption in scaffold design: (i) Data scarcity and heterogeneity: the field lacks open-access, standardised benchmark datasets; creating such databases would be the highest-leverage infrastructure investment the tissue engineering community could make. (ii) Model interpretability: black-box models provide limited mechanistic insight for rational design beyond the training distribution, and XAI adoption is not yet systematic. (iii) Regulatory acceptance: no current FDA or EMA guidance specifically addresses AI-generated scaffold designs, creating uncertainty about the evidentiary standards required for IND/IDE submissions based on ML-optimised materials. The reproducibility of scaffold optimization using AI is not consistent across different labs, indicating that standardized design software and databases will be key pre-requisites before ML scaffold design approaches can be adopted clinically.^246,247^

## 7. Conclusion

From the above critical analysis, the following combination shows promising translational potential; PLGA and nano-hydroxyapatite for bone regeneration due to its excellent osteoconductivity and regulatory approval, GelMA and CNF hybrid hydrogel for cartilage regeneration due to tunable mechanics and printability, collagen and electrospun PCL conduit for neural repair due to compatibility with current surgical procedures, chitosan and Ag nanoparticles for skin healing due to its intrinsic antimicrobial properties which help overcome infections, and finally electrospun PCL, collagen, and elastin composite for vascular regeneration due to closest compliance with native tissues. Despite this progress, three major obstacles must be overcome during the next five years: developing M2-polarizing (immune-neutral) interfaces without compromising their mechanical strength at physiological stress levels; industrialization of precise manufacturing technologies (*e.g.*, electrospinning, 3D bioprinting) for GMP-compliant personalized production; and development of a validated *in vitro-in vivo* correlation model to speed up regulatory approval. The absence of standard IVIVC requires that every scaffold undergoes complete preclinical evaluation, which increases both costs and timelines. The bare minimum test set is therefore required, which includes ISO 10993-5 cytotoxicity, ISO 10993-4 hemocompatibility, ISO 10993-6 implantation, complete nanoparticle analysis, mechanical tests according to tissue type, and accelerated degradation with subsequent toxicological analysis of degradation products. Moving into the future, the combination of 4D printing, stimuli-responsive materials, and design optimization through artificial intelligence, all in the context of an emerging and evolving regulatory framework, presents a viable path from laboratory breakthroughs to clinical application. Success in the years ahead will be contingent not only on the development of innovative new materials but more so on the discipline of translation, developing standard data sets and models of *in vitro-in vivo* correlation, and bringing together skills and knowledge in materials science, cellular biology, clinical medicine, and regulatory science.

## Conflicts of interest

There are no conflicts to declare.

## Data Availability

No new data were generated or analysed as part of this review.
